# Codon-specific *KRAS* mutations predict survival benefit of trifluridine/tipiracil in metastatic colorectal cancer

**DOI:** 10.1038/s41591-023-02240-8

**Published:** 2023-03-02

**Authors:** Joris van de Haar, Xuhui Ma, Salo N. Ooft, Pim W. van der Helm, Louisa R. Hoes, Sara Mainardi, David J. Pinato, Kristi Sun, Lisa Salvatore, Giampaolo Tortora, Ina Valeria Zurlo, Silvana Leo, Riccardo Giampieri, Rossana Berardi, Fabio Gelsomino, Valeria Merz, Federica Mazzuca, Lorenzo Antonuzzo, Gerardo Rosati, Chara Stavraka, Paul Ross, Maria Grazia Rodriquenz, Michele Pavarana, Carlo Messina, Timothy Iveson, Federica Zoratto, Anne Thomas, Elisabetta Fenocchio, Margherita Ratti, Ilaria Depetris, Massimiliano Cergnul, Cristina Morelli, Michela Libertini, Alessandro Parisi, Michele De Tursi, Nicoletta Zanaletti, Ornella Garrone, Janet Graham, Raffaella Longarini, Stefania Maria Gobba, Angelica Petrillo, Emiliano Tamburini, Nicla La Verde, Fausto Petrelli, Vincenzo Ricci, Lodewyk F. A. Wessels, Michele Ghidini, Alessio Cortellini, Emile E. Voest, Nicola Valeri

**Affiliations:** 1 Division of Molecular Oncology & Immunology, The Netherlands Cancer Institute, Amsterdam, the Netherlands; 2 Division of Molecular Carcinogenesis, The Netherlands Cancer Institute, Amsterdam, the Netherlands; 3 Oncode Institute, The Netherlands Cancer Institute, Amsterdam, the Netherlands; 4 Department of Surgery and Cancer, Imperial College London, Hammersmith Hospital, London, UK; 5 Division of Oncology, Department of Translational Medicine, University of Piemonte Orientale, Novara, Italy; 6 Imperial College Healthcare NHS Trust, London, UK; 7 Oncologia Medica, Comprehensive Cancer Center, Fondazione Policlinico Universitario Agostino Gemelli Istituto di Ricovero e Cura a Carattere Scientifico, Roma, Italy; 8 Oncologia Medica, Università Cattolica del Sacro Cuore, Roma, Italy; 9 Medical Oncology, ‘Vito Fazzi’ Hospital, Lecce, Italy; 10 Department of Oncology, Università Politecnica delle Marche, Azienda Ospedialiera Universitaria delle Marche, Ancona, Italy; 11 University Hospital of Modena, Modena, Italy; 12 Medical Oncology Unit, Santa Chiara Hospital, Trento, Italy; 13 Department of Clinical and Molecular Medicine, Sapienza University, Oncology Unit, Azienda Ospedialiera Universitaria Sant’Andrea, Rome, Italy; 14 Clinical Oncology Unit, Careggi University Hospital, Florence, Italy; 15 Department of Experimental and Clinical Medicine, University of Florence, Florence, Italy; 16 Medical Oncology Unit, S. Carlo Hospital, Potenza, Italy; 17 School of Cancer & Pharmaceutical Sciences, King’s College London, Guy’s Hospital, London, UK; 18 Department of Medical Oncology, Guy’s and St Thomas’ NHS Foundation Trust, London, UK; 19 Istituto di Ricovero e Cura a Carattere Scientifico Casa Sollievo della Sofferenza, San Giovanni Rotondo, Italy; 20 Oncology Unit, Azienda Ospedaliera Universitaria Integrata, Verona, Italy; 21 Oncology Unit, ARNAS Civico Di Cristina Benfratelli Hospital, Palermo, Italy; 22 University of Southampton, Southampton, UK; 23 Unità Operativa Complessa Oncologia, Ospedale Santa Maria Goretti Latina, Latina, Italy; 24 Leicester Cancer Research Centre, University of Leicester, Leicester Royal Infirmary, Leicester, UK; 25 Candiolo Cancer Institute FPO Istituto di Ricovero e Cura a Carattere Scientifico Candiolo, Candiolo, Italy; 26 Azienda Socio-Sanitaria Territoriale Cremona, Cremona, Italy; 27 Division of Medical Oncology, ASL TO4, Ospedale Civile di Ivrea, Ivrea, Italy; 28 Unità Operativa Oncologia Medica, Ospedale Civile di Legnano, Azienda Socio-Sanitaria Territoriale Ovest Milanese, Legnano, Italy; 29 Medical Oncology Unit, Department of Systems Medicine, Tor Vergata University Hospital, Rome, Italy; 30 Medical Oncology Unit, Brescia, Italy; 31 Department of Life, Health and Environmental Sciences, University of L’Aquila, L’Aquila, Italy; 32 Dipartimento di Tecnologie Innovative in Medicina & Odontoiatria, Università G. D’Annunzio, Chieti-Pescara, Chieti, Italy; 33 Experimental Clinical Abdominal Oncology Unit, Istituto Nazionale Tumori, Istituto di Ricovero e Cura a Carattere Scientifico, Fondazione G. Pascale, Naples, Italy; 34 Oncology Unit, Fondazione Istituto di Ricovero e Cura a Carattere Scientifico Ca’ Granda Ospedale Maggiore Policlinico, Milan, Italy; 35 Beatson West of Scotland Cancer Centre, Glasgow, UK; 36 San Gerardo Hospital, Monza, Italy; 37 Division of Clinical Oncology, Azienda Socio-Sanitaria Territoriale dei Sette Laghi Varese, Varese, Italy; 38 Medical Oncology Unit, Ospedale del Mare, Naples, Italy; 39 Oncology and Palliative Care Department, Tricase Hospital, Lecce, Italy; 40 Luigi Sacco Hospital-Polo Universitario, Azienda Socio-Sanitaria Territoriale Fatebenefratelli Sacco, Milan, Italy; 41 Oncology Unit, Azienda Socio-Sanitaria Territoriale Bergamo Ovest, Treviglio, Italy; 42 Medical Oncology Unit, Azienda Ospedaliera di Rilievo Nazionale ‘San Pio’, Benevento, Italy; 43 Faculty of Electrical Engineering, Mathematics and Computer Science, Delft University of Technology, Delft, the Netherlands; 44 Medical Oncology, Fondazione Policlinico Universitario Campus Bio-Medico, Rome, Italy; 45 Centre for Evolution and Cancer, The Institute of Cancer Research, London, UK

**Keywords:** Predictive markers, Cancer therapeutic resistance, Cancer genomics, Colorectal cancer, Chemotherapy

## Abstract

Genomics has greatly improved how patients with cancer are being treated; however, clinical-grade genomic biomarkers for chemotherapies are currently lacking. Using whole-genome analysis of 37 patients with metastatic colorectal cancer (mCRC) treated with the chemotherapy trifluridine/tipiracil (FTD/TPI), we identified *KRAS* codon G12 (*KRAS*^G12^) mutations as a potential biomarker of resistance. Next, we collected real-world data of 960 patients with mCRC receiving FTD/TPI and validated that *KRAS*^G12^ mutations were significantly associated with poor survival, also in analyses restricted to the *RAS*/*RAF* mutant subgroup. We next analyzed the data of the global, double-blind, placebo-controlled, phase 3 RECOURSE trial (*n* = 800 patients) and found that *KRAS*^G12^ mutations (*n* = 279) were predictive biomarkers for reduced overall survival (OS) benefit of FTD/TPI versus placebo (unadjusted interaction *P* = 0.0031, adjusted interaction *P* = 0.015). For patients with *KRAS*^G12^ mutations in the RECOURSE trial, OS was not prolonged with FTD/TPI versus placebo (*n* = 279; hazard ratio (HR) = 0.97; 95% confidence interval (CI) = 0.73–1.20; *P* = 0.85). In contrast, patients with *KRAS*^G13^ mutant tumors showed significantly improved OS with FTD/TPI versus placebo (*n* = 60; HR = 0.29; 95% CI = 0.15–0.55; *P* < 0.001). In isogenic cell lines and patient-derived organoids, *KRAS*^G12^ mutations were associated with increased resistance to FTD-based genotoxicity. In conclusion, these data show that *KRAS*^G12^ mutations are biomarkers for reduced OS benefit of FTD/TPI treatment, with potential implications for approximately 28% of patients with mCRC under consideration for treatment with FTD/TPI. Furthermore, our data suggest that genomics-based precision medicine may be possible for a subset of chemotherapies.

## Abstract (web summary)

A combination of real-world evidence and a reanalysis of phase 3 clinical trial data unveils *KRAS* codon G12 mutations as a biomarker of resistance to trifluridine/tipiracil in metastatic colorectal cancer.

## Main

Systemic anticancer therapy based on the chemotherapeutic agents 5-fluorouracil (5-FU)/capecitabine, oxaliplatin and irinotecan in combination with epidermal growth factor receptor (EGFR) or vascular endothelial growth factor inhibitors are the cornerstone of the treatment of metastatic colorectal cancer (mCRC)^1^. More recently, the chemotherapeutic drug trifluridine/tipiracil (FTD/TPI), a combination of trifluridine (FTD), a nucleoside analog, and tipiracil (TPI), a thymidine phosphorylase inhibitor, has been approved for patients with advanced, heavily pretreated mCRC^2–4^. Although durable responses to FTD/TPI have been observed in some patients with mCRC, the median overall survival (OS) benefit in the general population with mCRC is modest (1.8 months), highlighting the unmet need for patient selection^1,3,5–9^.

**Extended Data Fig. 1:**
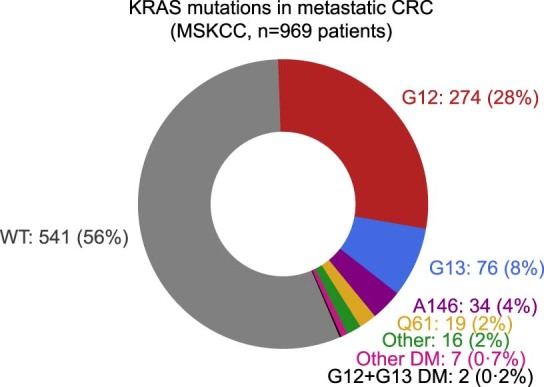
Codon-specific *KRAS* mutation frequencies in mCRC (MSKCC cohort^1^). WT: wild type; DM: double mutation.

Precision medicine is widely used to select patients for targeted therapies and immunotherapies for mCRC according to the presence or absence of genomic biomarkers. As such, the detection of *KRAS* hotspot mutations is a critical step in the diagnostic workup of mCRC as *RAS*/*RAF* mutations predict clinical resistance to EGFR-targeting antibodies^10–12^. Such *KRAS* mutations are found in 44% of patients with mCRC; these most frequently occur at codon G12 (*KRAS*^G12^; 28% of patients) or codon G13 (*KRAS*^G13^; 8% of patients) (Extended Data Fig. 1 and Supplementary Table 1)^13^. Although *KRAS*^G12^ and *KRAS*^G13^ mutations are regarded as a single entity in clinical practice guidelines, they have different biochemical properties^14,15^ and display tissue- and treatment-specific mutational patterns^16^.

**Extended Data Fig. 2:**
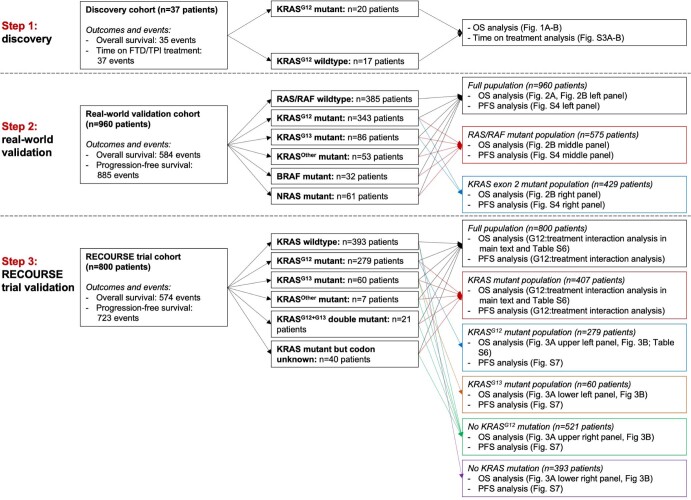
Overview of the study and the cohorts used in the analyses.

In this study, given the lack of genomic biomarkers and the limited clinical benefit of FTD/TPI in unselected patients with mCRC, we harnessed the power of whole-genome somatic profiles coupled with patient outcomes to identify biomarkers of response and resistance to FTD/TPI. Key findings were then validated in a real-world cohort of FTD/TPI-treated patients with mCRC (*n* = 960) and in the double-blind, placebo-controlled, phase 3 RECOURSE trial (*n* = 800; study overview shown in Extended Data Fig. 2).

## Results

### *KRAS*^G12^ mutations as potential biomarkers for FTD/TPI treatment

**Fig. 1:**
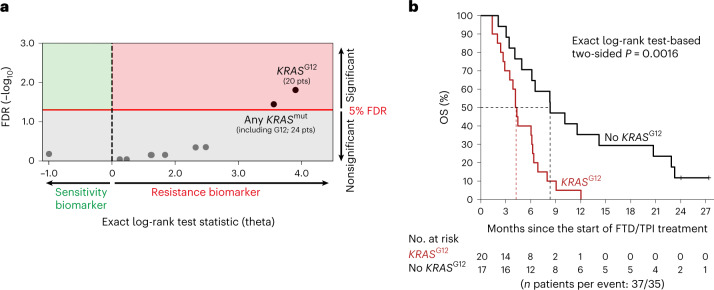
Discovery of *KRAS*^G12^ mutation status as potential biomarker of outcome of FTD/TPI treatment in mCRC. **a**, Dot plot showing the associations of candidate genomic biomarkers to OS on FTD/TPI treatment in the discovery cohort (*n* = 37 patients). The exact log-rank test statistic (theta) for the death of patients with the candidate biomarker versus those without is plotted against the Benjamini–Hochberg-corrected FDR. The red line indicates the 5% FDR significance threshold. **b**, A Kaplan–Meier curve of OS in the discovery cohort for patients without (black) or with (red) a *KRAS*^G12^ mutation. Censoring events are indicated by vertical bars on the corresponding curve. The dotted lines indicate the median OS. The table underneath the plot denotes the numbers at risk. The exact log-rank test-based two-sided *P* is shown. FDR, false discovery rate; OS, overall survival.

**Extended Data Fig. 3:**
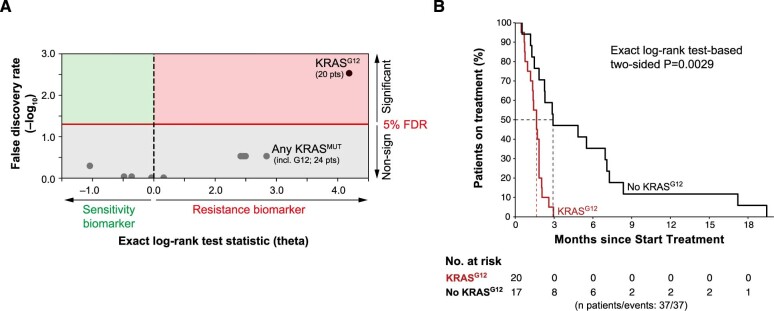
Discovery of KRAS^G12^ mutation status as potential biomarker of outcome of FTD/TPI treatment in mCRC. **a** dot plot showing the associations of candidate genomic biomarkers to time on FTD/TPI treatment in the discovery cohort (n = 37). The exact log-rank test statistic (theta) for treatment discontinuation for patients with the candidate biomarker versus those without is plotted against the Benjamini-Hochberg-corrected false discovery rate (FDR). The red line indicates the 5% FDR significance threshold. **b** A Kaplan-Meier curve of time on treatment in the discovery cohort, for patients without (black) or with (red) a KRAS^G12^ mutation. Censoring events are indicated by vertical bars on the corresponding curve. Dotted lines indicate the median overall survival. The table underneath the plot denotes the numbers at risk. The exact log-rank test-based two-sided *P* value is shown.

We first performed whole-genome analysis of a real-world discovery cohort that consisted of 37 patients with mCRC from the publicly available Hartwig Medical Foundation database^17^, who received FTD/TPI treatment in a standard-of-care setting in 13 hospitals across the Netherlands (Hartwig Medical Foundation (HMF) cohort; Supplementary Table 2). In accordance with late-stage disease, the median OS was relatively short, that is, 6.1 months (95% confidence interval (CI) = 4.2– 8.3). Ten genomic drivers occurred in at least five patients and were tested as candidate biomarkers for OS (Supplementary Table 3 and Methods). After correction for multiple-hypothesis testing, *KRAS*^G12^ status was most significantly associated with reduced OS (exact log-rank test-based two-sided *P* = 0.0016; Benjamini–Hochberg false discovery rate (FDR) = 0.016; threshold for significance, FDR < 0.05; Fig. 1a,b and Supplementary Table 4). Besides 20 patients with *KRAS*^G12^ mutations, the cohort also included four patients with *KRAS* mutations at other codons. Consideration of all *KRAS* mutations combined in a codon-agnostic manner diluted the observed effect (Fig. 1a,b and Supplementary Table 4). Similar results were obtained when time on FTD/TPI treatment was used as the end point (Extended Data Fig. 3 and Supplementary Table 5). Based on these hypothesis-generating results, we wondered if *KRAS*^G12^ mutation status could be a determinant of FTD/TPI treatment outcome in mCRC.

### *KRAS* mutations and real-world survival on FTD/TPI treatment

We next collected real-world data of 960 patients with mCRC who were treated with FTD/TPI in 36 centers across Italy and the UK (Supplementary Tables 6 and 7). Based on routine diagnostics (largely performed at diagnosis), the cohort contained 385 patients with *RAS*/*RAF* wild-type (WT) tumors, 343 patients with *KRAS*^G12^ mutations, 86 patients with *KRAS*^G13^ mutations, 53 patients with *KRAS* mutations at codons other than G12 or G13 (*KRAS*^other^), 32 patients with *BRAF* mutations and 61 patients with *NRAS* mutations. In the full population, patients with *KRAS*^G12^ mutations had more frequent right-sided disease and more recent diagnoses of metastatic disease (Table 1). Importantly, these factors were well balanced when patients with *KRAS*^G12^ mutations were compared to patients with other *RAS*/*RAF* mutations, or specifically to those with hotspot mutations affecting the directly adjacent codon *KRAS*^G13^, whereas this latter subgroup had relatively good performance status (Table 1).

**Table 1:** Baseline characteristics of patients in the real-world validation cohort, stratified according to codon-specific *RAS*/*RAF* mutation status

Characteristic	Overall (*n* = 960)	*KRAS*^G12^ (*n* = 343)	*KRAS*^G13^ (*n* = 86)	*KRAS*^O^^ther^ (*n* = 53)	*BRAF*^mut^ (*n* = 32)	*NRAS*^mut^ (*n* = 61)	*RAS*/*RAF*^WT^ (*n* = 385)^b^	Overall (*n* = 960) G12 versus no G12	*RAS*/*RAF*^mut^(*n* = 575) G12 versus no G12	*KRAS*^exon_2_mut^ (*n* = 429)^a^ G12 versus G13
**Age**										
Median (IQR)	64 (56–71)	65 (57–72)	63 (56–72)	64 (60–69)	66 (57–72)	65 (56–73)	64 (55–71)	*P* = 0.44	*P* = 0.92	*P* = 0.70
<65	484 (50)	168 (49)	46 (53)	28 (53)	16 (50)	28 (46)	198 (51)	*P* = 0.54	*P* = 0.78	*P* = 0.47
≥65	476 (50)	175 (51)	40 (47)	25 (47)	16 (50)	33 (54)	187 (49)			
**Sex**										
Female	390 (41)	140 (41)	45 (52)	22 (42)	5 (16)	23 (38)	155 (40)	*P* = 0.95	*P* = 1.0	*P* = 0.067
Male	570 (59)	203 (59)	41 (48)	31 (58)	27 (84)	38 (62)	230 (60)			
**Country**										
Italy	827 (86)	289 (84)	78 (91)	47 (89)	28 (88)	54 (89)	331 (86)	*P* = 0.21	*P* = 0.11	*P* = 0.17
UK	133 (14)	54 (16)	8 (9)	6 (11)	4 (12)	7 (11)	54 (14)			
**ECOG performance score**										
0–1	820 (85)	292 (85)	83 (97)	42 (79)	23 (72)	53 (87)	327 (85)	*P* = 0.85	*P* = 0.28	***P*** **=** **0.0031**
≥2	140 (15)	51 (15)	3 (3)	11 (21)	9 (28)	8 (13)	58 (15)			
**Primary site of disease**										
Colon	696 (71)	262 (76)	62 (72)	37 (70)	24 (75)	42 (69)	269 (70)	*P* = 0.050	*P* = 0.24	*P* = 0.40
Rectum	264 (28)	81 (24)	24 (28)	16 (30)	8 (25)	19 (31)	116 (30)			
**Sidedness**										
Left	661 (69)	205 (60)	53 (62)	34 (64)	18 (56)	43 (70)	308 (80)	***P*** < **0.001**	P = 0.40	*P* = 0.81
Right^c^	299 (31)	138 (40)	33 (38)	19 (36)	14 (44)	18 (30)	77 (20)			
**Time from diagnosis of metastases**										
<18 months	275 (28)	117 (34)	27 (31)	19 (36)	16 (50)	20 (33)	76 (20)	***P*** = **0.0073**	*P* = 0.77	*P* = 0.70
≥18 months	678 (71)	225 (66)	59 (69)	34 (64)	15 (47)	40 (66)	305 (79)			
Unknown	7 (1)	1 (0)	0 (0)	0 (0)	1 (3)	1 (2)	4 (1)			
**Previous surgery**										
No	229 (24)	84 (24)	15 (17)	11 (21)	12 (38)	19 (31)	88 (23)	*P* = 0.75	*P* = 0.75	*P* = 0.20
Yes	729 (76)	259 (76)	70 (81)	42 (79)	20 (62)	42 (69)	296 (77)			
Unknown	2 (0)	0 (0)	1 (1)	0 (0)	0 (0)	0 (0)	1 (0)			
**Peritoneal disease at the start of FTD/TPI treatment**										
No	604 (63)	203 (59)	56 (65)	32 (60)	17 (53)	38 (62)	258 (67)	*P* = 0.070	*P* = 0.51	*P* = 0.33
Yes	355 (37)	140 (41)	30 (35)	21 (40)	15 (47)	22 (36)	127 (33)			
Unknown	1 (0)	0 (0)	0 (0)	0 (0)	0 (0)	1 (2)	0 (0)			
**MMR status**										
Proficient	563 (59)	197 (56)	59 (69)	34 (64)	20 (62)	38 (62)	215 (56)	*P* = 0.55	*P* = 1.0	*P* = 0.69
Deficient	28 (3)	8 (2)	1 (1)	4 (8)	3 (9)	0 (0)	12 (3)			
Unknown	369 (38)	138 (40)	26 (30)	15 (28)	9 (28)	23 (38)	158 (41)			

^a^*KRAS*^exon_2_mut^: patients with *KRAS* exon 2 mutations (*KRAS*^G12^ and *KRAS*^G13^). ^b^*RAS*/*RAF*^WT^: patients without *KRAS*, *NRAS* or *BRAF* mutations. ^c^Right-sided disease, including five patients with multiple primary tumors at both sides. Two-sided *P* values (bold for *P* < 0.05) were used for comparisons between subgroups as indicated by the headers, using a Fisher’s exact test for categorical variables (excluding unknowns) and a Wilcoxon rank-sum test for continuous age. IQR, interquartile range.

**Fig. 2:**
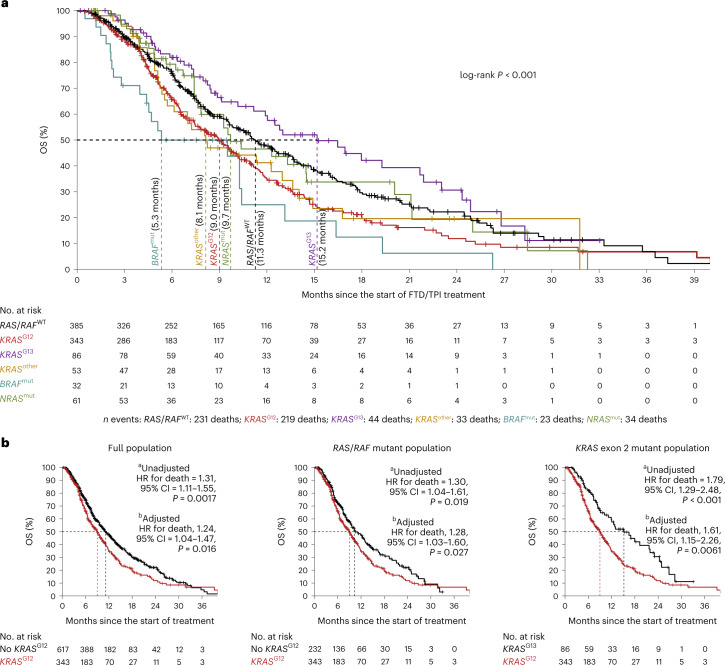
Associations of *RAS*/*RAF* mutations with the OS of 960 patients with mCRC receiving FTD/TPI treatment in a real-world setting. **a**, Kaplan–Meier curve of OS in the full population, stratified according to *RAS*/*RAF* mutations, as indicated by the colors (see the table underneath the plot for the color coding used for each *RAS*/*RAF* mutation category). Censoring events are indicated by vertical bars on the corresponding curve. The dotted lines and corresponding annotation indicate the subgroup-specific median OS. The table underneath the plot denotes the numbers at risk. The two-sided log-rank test-based *P* value is shown. **b**, Kaplan–Meier curves of OS in the full population (left), *RAS*/*RAF* mutant population (middle) and *KRAS*^exon_2_mut^ population (right), stratified according to the presence (red) or absence (black) of a *KRAS*^G12^ mutation. Censoring events are indicated by vertical bars on the corresponding curve. The dotted lines indicate the subgroup-specific median OS. The table underneath each plot denotes the numbers at risk. Two-sided Wald test-based *P* values are shown. ^a^Unadjusted by univariate Cox regression. ^b^Adjusted by stratified, multivariate Cox regression, adjusted for eight baseline characteristics (Methods). Note that all Cox regression models passed the proportional-hazards assumption. OS, overall survival.

**Extended Data Fig. 4:**
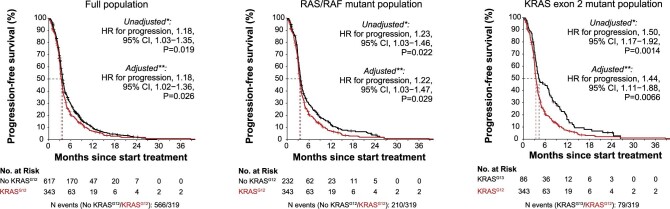
Associations of KRAS^G12^ mutations with progression-free survival of 960 patients with mCRC receiving FTD/TPI treatment in a real-world setting. Kaplan-Meier curves of progression-free survival in the full population (left), RAS/RAF mutant population (middle), and *KRAS* exon 2 mutant population (right), stratified based on the presence (red) or absence (black) of a KRAS^G12^ mutation. Censoring events are indicated by vertical bars on the corresponding curve. Dotted lines indicate the median overall survival. The table underneath each plot denotes the numbers at risk. Two-sided Wald test-based *P* values are shown. *Unadjusted: By univariate Cox regression. **Adjusted: By stratified, multivariate Cox regression, adjusted for eight baseline characteristics (see methods). Note that none of the Cox regression models significantly violated the proportional hazards assumption, despite crossing survival curves.

In the real-world validation cohort, codon-specific *RAS*/*RAF* mutations were significantly associated with clear differences in OS on treatment with FTD/TPI (log-rank *P* < 0.001; Fig. 2a). Again, *KRAS*^G12^ mutations were significantly associated with poor OS, with a similar effect in the population as a whole (unadjusted hazard ratio (HR) for death = 1.31; 95% CI = 1.11–1.55; *P* = 0.0017; adjusted HR for death = 1.24; 95% CI = 1.04–1.47, *P* = 0.016; Fig. 2b, left panel), as in the *RAS*/*RAF* mutant subpopulation (unadjusted HR for death = 1.30; 95% CI, 1.04–1.61, *P* = 0.019; adjusted HR for death, 1.28; 95% CI 1.03–1.60, *P* = 0.027; Fig. 2b, middle panel). Notably, the OS of patients with *KRAS*^G12^ mutations was also poor as compared with patients with *KRAS*^G13^ mutations (unadjusted HR for death = 1.79; 95% CI = 1.29–2.48; *P* < 0.001; adjusted HR for death = 1.61; 95% CI = 1.15–2.26, *P* = 0.0061; Fig. 2b, right panel). Similar results were obtained when the analysis was based on progression-free survival (PFS) (Extended Data Fig. 4). Patients with *KRAS*^G12^ mutations did not show significantly shorter OS than patients in any of the other, smaller *RAS*/*RAF* mutant subgroups (those with *KRAS*^other^, *BRAF* or *NRAS* mutations; statistics not shown). Together, this independent validation confirmed that patients with *KRAS*^G12^ mutant mCRC have relatively poor OS on treatment with FTD/TPI. Furthermore, the clear OS difference between patients with *KRAS*^G12^ and *KRAS*^G13^ mutations underwrites the rationale for considering *KRAS* mutations in a codon-specific manner.

### *KRAS* mutations and survival in the RECOURSE trial

To further strengthen our findings and investigate if our observations were based on prognostic or predictive effects, we analyzed the data of a large, independent, placebo-controlled clinical cohort, the RECOURSE trial^3^. Briefly, this international, randomized, double-blind, placebo-controlled, phase 3 study assigned 800 heavily pretreated patients with mCRC to receive either FTD/TPI or placebo in a 2:1 ratio. Based on routine diagnostics (largely performed at diagnosis), approximately half of the patients (*n* = 393) were *KRAS*^WT^, whereas the other half (*n* = 407) were *KRAS* mutant. In this study, *KRAS* mutation status (mutated yes/no) was not significantly associated with reduced OS or PFS benefit of FTD/TPI versus placebo; however, codon-specific analyses were not performed^3,4^.

Codon-specific mutational status was available for 367 out of 407 (90%) patients with *KRAS*-mutated tumors in the RECOURSE trial. Of these, 279 (76%) had *KRAS*^G12^ mutations, 60 (16%) had *KRAS*^G13^ mutations, 21 (5.7%) were reported to have *KRAS*^G12/G13^ double mutations (largely due to the use of analytical methods that could not discriminate between the two codons) and 7 (1.9%) had other mutations. (The true percentage of patients with other mutations was probably higher because their assessment was only broadly implemented later^11^.) Throughout our analyses, we considered patients with *KRAS*^G12/G13^ double mutations as a distinct subgroup.

The prespecified baseline characteristics of the RECOURSE trial were well balanced between the FTD/TPI and placebo arms in *KRAS*^G12^ mutant, *KRAS*^G13^ mutant and *KRAS*^WT^ subgroups (Table 2), with some exceptions; patients whose tumors harbored a *KRAS* mutation generally had more recent diagnoses of metastatic disease, were less heavily pretreated and were more frequently refractory to fluoropyrimidine as part of the last previous regimen (Table 2). Importantly, all these factors were balanced between the *KRAS*^G12^ and *KRAS*^G13^ mutant populations. Between these two groups, the only significant difference was that patients with *KRAS*^G13^ mutations originated less often from Japan (Table 2).

**Table 2:** Baseline characteristics of patients in the RECOURSE trial intention-to-treat population, stratified according to codon-specific *KRAS* mutation status

Characteristic	*KRAS*^G12^			*KRAS*^G13^			*KRAS*^WT^			G12 versus G13	G12 versus WT	G13 versus WT
	FTD/TPI (*n* = 185)	*P*	Placebo (*n* = 94)	FTD/TPI (*n* = 40)	*P*	Placebo (*n* = 20)	FTD/TPI (*n* = 262)	*P*	Placebo (*n* = 131)			
**Age**												
<65	114 (62)	*P* = 0.44	53 (56)	23 (57)	*P* = 0.17	7 (35)	138 (53)	*P* = 0.52	74 (56)	*P* = 0.19	*P* = 0.13	*P* = 0.58
≥65	71 (38)		41 (44)	17 (43)		13 (65)	124 (47)		57 (44)			
**Sex**												
Female	78 (42)	*P* = 0.80	38 (40)	19 (48)	*P* = 0.42	7 (35)	93 (35)	*P* = 0.66	50 (38)	*P* = 0.86	*P* = 0.20	*P* = 0.32
Male	107 (58)		56 (60)	21 (53)		13 (65)	169 (65)		81 (62)			
**Region**												
Japan	63 (34)	*P* = 0.19	40 (43)	9 (23)	*P* = 1.00	4 (20)	94 (36)	*P* = 0.31	40 (31)	***P*** = **0.025**	*P* = 0.46	*P* = 0.075
USA, Europe and Australia	122 (66)		54 (57)	31 (78)		16 (80)	168 (64)		91 (69)			
**ECOG**												
0	110 (59)	*P* = 0.61	59 (63)	23 (57)	*P* = 1.00	11 (55)	151 (58)	***P*** = **0.042**	61 (47)	*P* = 0.66	*P* = 0.097	*P* = 0.78
1	75 (41)		35 (37)	17 (43)		9 (45)	111 (42)		70 (53)			
**Primary site of disease**												
Colon	124 (67)	*P* = 0.19	55 (59)	23 (57)	*P* = 0.15	16 (80)	157 (60)	*P* = 0.83	77 (59)	*P* = 1.0	*P* = 0.23	*P* = 0.48
Rectum	61 (33)		39 (41)	17 (43)		4 (20)	105 (40)		54 (41)			
**Time from diagnosis of metastases**												
<18 months	51 (28)	*P* = 0.89	25 (27)	13 (33)	*P* = 0.58	8 (40)	39 (15)	*P* = 0.88	18 (14)	*P* = 0.27	***P*** < **0.001**	***P*** < **0.001**
≥18 months	134 (72)		69 (73)	27 (68)		12 (60)	223 (85)		113 (86)			
**No. of previous regimens**												
2	52 (28)	*P* = 0.95	26 (28)	13 (33)	*P* = 0.76	8 (40)	25 (10)	*P* = 0.37	8 (6)	*P* = 0.34	***P*** < **0.001**	***P*** < **0.001**
3	50 (27)		24 (26)	7 (18)		4 (20)	51 (19)		22 (17)			
≥4	83 (45)		44 (47)	20 (50)		8 (40)	186 (71)		101 (77)			
**Refractory to fluoropyrimidine as part of last previous regimen**												
Yes	135 (73)	*P* = 0.56	72 (77)	30 (75)	*P* = 0.54	13 (65)	117 (45)	*P* = 0.19	49 (37)	*P* = 0.75	***P*** < **0.001**	***P*** < **0.001**
No	50 (27)		22 (23)	10 (25)		7 (35)	145 (55)		82 (63)			
**Prior use of regorafenib**												
Yes	31 (17)	*P* = 0.60	13 (14)	5 (13)	*P* = 1.00	3 (15)	41 (16)	*P* = 0.12	29 (22)	*P* = 0.84	*P* = 0.53	*P* = 0.47
No	154 (83)		81 (86)	35 (88)		17 (85)	221 (84)		102 (78)			
**No. of metastatic sites**												
1–2	109 (59)	*P* = 0.30	62 (66)	22 (55)	*P* = 0.79	10 (50)	166 (63)	*P* = 0.064	70 (53)	*P* = 0.31	*P* = 0.75	*P* = 0.33
≥3	76 (41)		32 (34)	18 (45)		10 (50)	96 (37)		61 (47)			

Two-sided *P* values (bold for *P* < 0.05) are for comparisons between codon-specific *KRAS* mutation status-based subgroups, using Fisher’s exact test for categorical variables with two classes and chi-squared test for categorical variables with more than two classes. ECOG, Eastern Cooperative Oncology Group.

**Extended Data Fig. 5:**
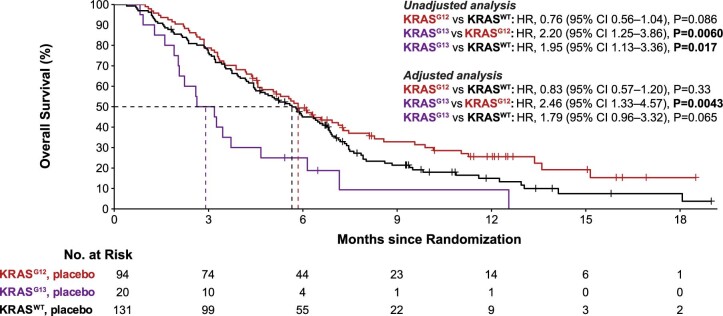
Kaplan-Meier curves of overall survival of KRAS^G12^-mutated, KRAS^G13^-mutated and KRAS^WT^ patients in the placebo arm of the RECOURSE trial. Censoring events are indicated by vertical bars on the corresponding curve. Dotted lines and corresponding annotation indicate the median overall survival. The table underneath the plot denotes the numbers at risk. Cox regression-based hazard ratio (HR), 95% confidence interval (CI) and two-sided Wald test-based *P* values are plotted for pairwise comparisons.

To understand the prognostic effects of codon-specific *KRAS* mutations in the trial population, we first analyzed OS in the placebo arm (Extended Data Fig. 5). This showed that patients with the *KRAS*^G12^ and *KRAS*^WT^ mutants had similar OS. Interestingly, placebo-treated patients with *KRAS*^G13^ mutations (the other main *KRAS* mutant subgroup in the study) had a remarkably shorter OS than those with *KRAS*^G12^ mutations (median OS *KRAS*^G13^ mutants: 2.9 months, 95% CI = 2.1–6.1 months versus median OS *KRAS*^G12^ mutants: 5.8 months, 95% CI = 4.7–7.3; HR = 2.20; 95% CI = 1.25–3.86; *P* = 0.0060; Extended Data Fig. 5), which held after adjustment for the ten baseline characteristics (HR = 2.46; 95% CI = 1.33–4.57; *P* = 0.0043; Extended Data Fig. 5). In the placebo arm, patients with *KRAS*^G13^ mutant tumors also had shorter OS than those with *KRAS*^WT^ tumors, which was statistically significant in unadjusted analysis (HR = 1.95; 95% CI = 1.13–3.36; *P* = 0.017; Extended Data Fig. 5), but did not attain statistical significance in the adjusted analysis (HR = 1.79; 95% CI = 0.96–3.32; *P* = 0.065; Extended Data Fig. 5). Taken together, these analyses indicate that *KRAS*^G12^ mutations are not associated with poor prognosis in late-stage mCRC.

**Fig. 3:**
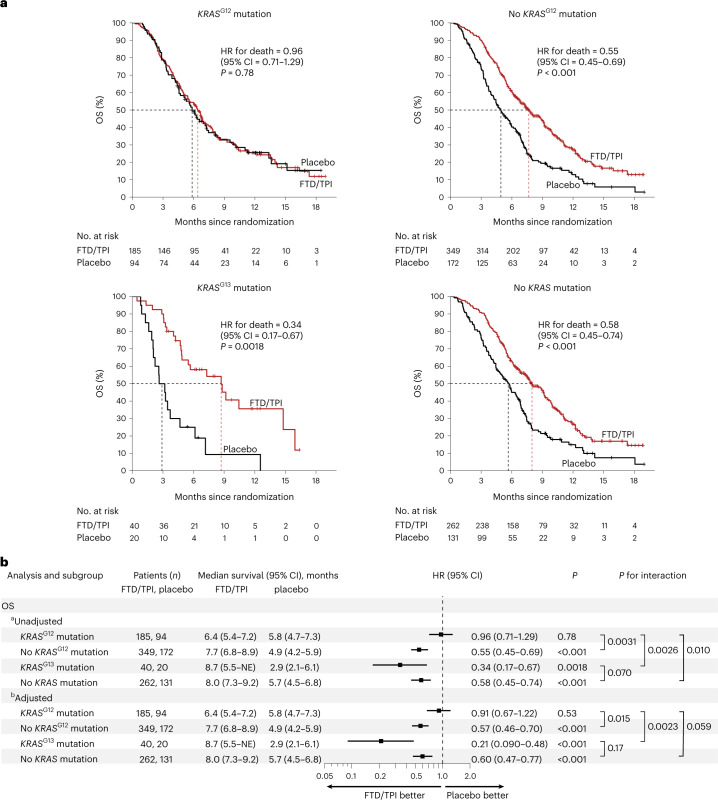
*KRAS* mutations and OS benefit of FTD/TPI versus placebo in the RECOURSE trial. **a**, Kaplan–Meier curves of OS with FTD/TPI (red) or placebo (black) for patients with *KRAS*^G12^ mutations (upper left panel), without *KRAS*^G12^ mutations (upper right panel), with *KRAS*^G13^ mutations (lower left panel) and without *KRAS* mutations (lower right panel). Censoring events are indicated by vertical bars on the corresponding curve. The dotted lines indicate the median OS. The table underneath each plot denotes the numbers at risk. Two-sided Wald test-based *P* values are shown. **b**, Forest plot of HRs for death and 95% CIs for patients treated with FTD/TPI versus placebo, subgrouped according to codon-specific *KRAS* mutation status. Two-sided Wald test-based *P* values for interaction (as calculated using Cox regression) indicate if the OS benefit of FTD/TPI treatment versus placebo was significantly different between subgroups, for which pairwise comparisons are indicated by the square brackets. ^a^Unadjusted: stratified for two stratification factors of the trial (time from diagnosis of metastases (<18 versus ≥18 months) and region (Japan versus USA, Europe and Australia)). ^b^Adjusted: adjusted by the two stratification factors used in unadjusted analysis plus eight additional baseline characteristics (Methods). Note that all Cox regression models passed the proportional-hazards assumption. NE, not estimated; OS, overall survival.

**Extended Data Fig. 6:**
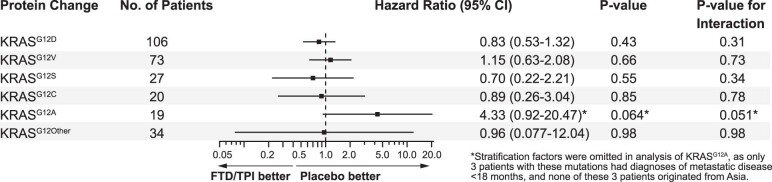
Forest plot of hazard ratios and 95% CI for overall survival with FTD/TPI versus placebo, for the KRAS^G12^-mutated population in amino acid change-based subgroups. The five most frequent KRAS^G12^ amino acid changes are shown as individual subgroups. KRAS^G12Other^ comprises all patients with KRAS^G12^ mutations that induce amino acid changes other than these five most frequent changes. We excluded 21 patients with KRAS^G12^ mutations for which data on the amino acid change was missing. Two-sided Wald test-based *P* values for interaction (as calculated using Cox regression) indicate if the survival benefit of FTD/TPI treatment versus placebo was significantly different for a specific subgroup, as compared to all patients with other KRAS^G12^ mutations.

We then studied if *KRAS*^G12^ mutations were predictive biomarkers for reduced OS benefit of FTD/TPI in the RECOURSE trial. In the *KRAS*^G12^ mutant population (*n* = 279 patients), OS was not prolonged with FTD/TPI versus placebo (HR = 0.96; 95% CI = 0.71–1.29; *P* = 0.78; Fig. 3a, upper left). Within the full study population (*n* = 800 patients), *KRAS*^G12^ mutations were significantly associated with a reduced OS benefit of FTD/TPI versus placebo (unadjusted interaction *P* = 0.0031; adjusted interaction *P* = 0.015; Fig. 3b; full regression model fits shown in Supplementary Table 8). In analyses restricted to the subgroup with *KRAS* mutations (*n* = 407 patients), *KRAS*^G12^ mutations were also significantly associated with reduced OS benefit of FTD/TPI versus placebo (unadjusted interaction *P* = 0.0091; adjusted interaction *P* = 0.0037; full regression model fits shown in Supplementary Table 8). Further stratification of patients with *KRAS*^G12^ mutations according to different amino acid changes did not provide evidence of OS benefit with FTD/TPI versus placebo in any subgroup (Extended Data Fig. 6). Taken together, these data demonstrate that FTD/TPI treatment did not lead to a clinically relevant prolongation of OS in patients with mCRC with *KRAS*^G12^ mutations in the RECOURSE trial.

When patients whose tumors harbored a *KRAS*^G12^ mutation were excluded from the analysis, FTD/TPI resulted in a pronounced OS benefit over placebo (*n* = 521; HR = 0.55; 95% CI = 0.45–0.69; *P* < 0.001; Fig. 3a, upper right), with a median OS benefit of 2.7 months in this subgroup (versus 1.8 months in the full population, as reported by Mayer et al.^3^).

Next, we analyzed the treatment effect of FTD/TPI in patients with *KRAS*^G13^ mutant tumors. In sharp contrast to the *KRAS*^G12^ mutant population, patients with the *KRAS*^G13^ mutation showed a clear OS benefit in the FTD/TPI arm versus the placebo arm (HR = 0.34; 95% CI = 0.17–0.67; *P* = 0.0018; Fig. 3a, lower left). This remained significant in the adjusted analysis (HR = 0.21; 95% CI = 0.090–0.48; *P* < 0.001; Fig. 3b). The median OS was three times longer in the FTD/TPI arm versus the placebo arm (8.7 versus 2.9 months; Fig. 3). The OS benefit of FTD/TPI treatment was significantly more pronounced in patients with *KRAS*^G13^-mutated mCRC versus those with *KRAS*^G12^-mutated disease (unadjusted interaction *P* = 0.0026; adjusted interaction *P* = 0.0023; Fig. 3b; the full regression model fits are shown in Supplementary Table 8). Thus, *KRAS*^G13^ mutations marked patients with clear OS benefit from FTD/TPI treatment.

**Extended Data Fig. 7:**
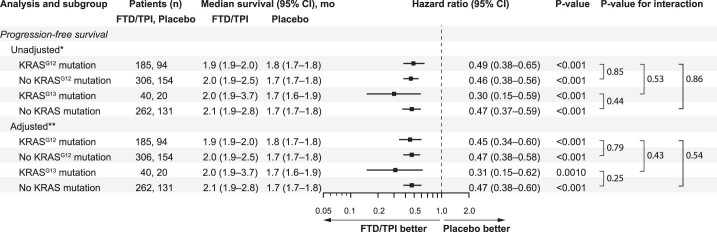
Progression-free survival in codon-specific *KRAS* mutation-based subgroups in the RECOURSE trial. Forest plot of hazard ratios for progression or death and 95% CI, stratified based on codon-specific *KRAS* mutation status. Two-sided Wald test-based *P* values for interaction (as calculated using Cox regression) indicate if the survival benefit of FTD/TPI treatment versus placebo was significantly different between subgroups, for which pairwise comparisons are indicated by the square brackets. *Unadjusted: stratified for two stratification factors of the trial (time from diagnosis of metastases [<18 mo versus ≥18 mo] and region [Japan versus United States, Europe, and Australia]). **Adjusted: adjusted by the two stratification factors used in unadjusted analysis, plus eight additional baseline characteristics (see methods). NE: not estimable.

We then assessed PFS in *KRAS* codon-specific subgroups of the RECOURSE trial. A minimal PFS benefit of FTD/TPI versus placebo was observed in all three subgroups (median PFS benefit 0.1, 0.3 and 0.3 months for patients with *KRAS*^G12^, *KRAS*^G13^ and *KRAS*^WT^ mutations, respectively), which did not significantly differ among these subpopulations (interactions nonsignificant for all pairwise comparisons; Extended Data Fig. 7).

### *KRAS*^G12^ mutations and FTD/TPI resistance in vitro

**Fig. 4:**
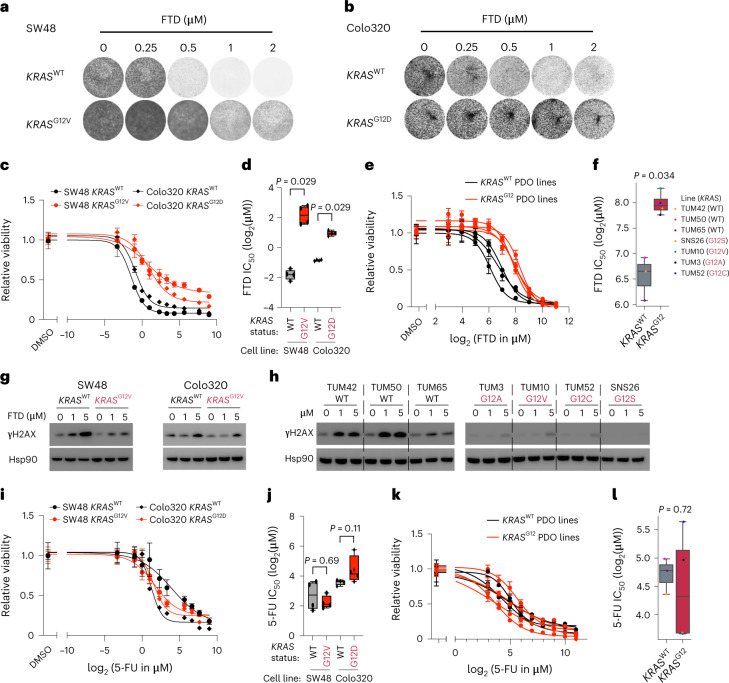
*KRAS*^G12^ mutations and in vitro resistance to FTD in isogenic cell lines and PDOs of mCRC. **a**, Colony formation assay for *KRAS*^WT^ and *KRAS*^G12V^ SW48 colorectal cancer cell lines after 2 weeks’ exposure to a concentration range of FTD in vitro. **b**, As in **a**, but for *KRAS*^WT^ and *KRAS*^G12D^ isogenic Colo320 CRC cell lines. **c**, Dose–response curves of *KRAS*^WT^ (black) and *KRAS*^G12^ (red) isogenic SW48 (dots) or Colo320 (diamonds) CRC cell lines exposed to a concentration range of FTD in vitro. The dots and error bars represent the mean and s.d. among four biological replicates at the tested concentrations, respectively. **d**, Half-maximal inhibitory concentrations (IC_50_; log_2_) for FTD of *KRAS*^WT^ and *KRAS*^G12^ isogenic SW48 or Colo320 CRC cell lines, as indicated on the *x* axis. Data are plotted for four biological replicates. The box center lines, box ranges, whiskers and dots indicate the medians, quartiles, 1.5 times the IQR and data points of individual experiments (biological replicates; *n* = 4 for each line), respectively. The two-sided Wilcoxon rank-sum test-based *P* value is shown. **e**, Dose–response curves of mCRC PDOs harboring WT *KRAS* (black; *n* = 3) or different *KRAS*^G12^ mutations (red; *n* = 4) exposed to FTD in vitro. The dots and error bars represent the mean and s.d. at the tested concentrations, respectively. **f**, IC_50_ (log_2_) for FTD of *KRAS*^WT^ (black; *n* = 3) and *KRAS*^G12^ (red; *n* = 4) mCRC PDOs. The box center lines, box ranges, whiskers and dots indicate the medians, quartiles, 1.5 times the IQR and data points of individual organoid lines (see legend), respectively. The two-sided Wilcoxon rank-sum test-based *P* value is shown. **g**, Representative western blot of the DNA damage marker γH2AX on treatment of *KRAS*^WT^ (black) and *KRAS*^G12^ (red) SW48 (left) and Colo320 (right) cells with FTD at increasing concentrations. Hsp90 was used as a loading control. Data were confirmed in three and two biological replicates for SW48 and Colo320, respectively. **h**, As in **g**, but for mCRC PDOs harboring *KRAS*^WT^ (black, left) or different *KRAS*^G12^ mutations (red, right). The left and right panels were exposed together (Source Data 1). Data were confirmed in three biological replicates. **i**, As in **c** but for 5-FU. **j**, As in **d** but for 5-FU. **k**, As in **e** but for 5-FU. **l,** As in **f** but for 5-FU. Source data

**Extended Data Fig. 8:**
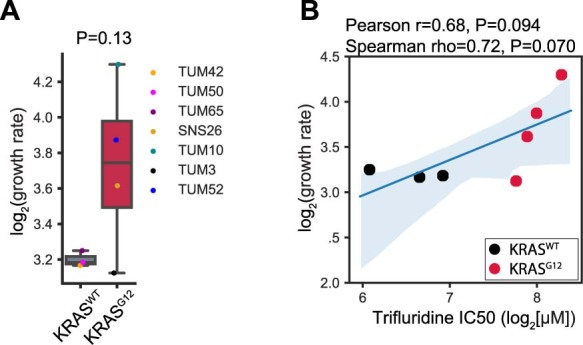
Growth rate of patient-derived mCRC organoids versus KRAS-based subgroup or *in vitro* trifluridine sensitivity. **a** The (log_2_) *in vitro* growth rate of patient-derived mCRC organoids in the untreated condition is plotted as stratified per *KRAS* mutation-based subgroup. Box center lines, box ranges, whiskers, and dots indicate medians, quartiles, 1.5 interquartile ranges, and data points of individual organoid lines, respectively. Colors of dots denote the organoid line, as shown in the legend. Two-sided two sample t-test-based *P* value is shown. **b** Half maximal inhibitory concentrations (IC50; log_2_) for trifluridine of KRAS^WT^ (black; n = 3) and KRAS^G12^ (red; n = 4) organoid lines (x-axis), is plotted against the (log_2_) *in vitro* growth rate of patient-derived mCRC organoids in the untreated condition (y-axis). The linear fit and bootstrapping-obtained 95% confidence interval of the regression estimate is denoted in dark and light blue, respectively. Pearson and Spearman correlation coefficients and corresponding *P* values are shown.

Finally, we aimed to replicate these findings in vitro using isogenic cell lines and mCRC patient-derived organoids (PDOs) (*n* = 7; Supplementary Table 9). *KRAS*^G12^ mutation knock in significantly reduced responsiveness to FTD (the cytotoxic component of FTD/TPI) in two colorectal cancer cell line models, SW48 and Colo320 (two-sided Wilcoxon rank-sum-based *P* = 0.029 for both models; Fig. 4a–d). The parental models are *KRAS*^WT^ and do not harbor other frequent mCRC oncogenic drivers like mutations in *NRAS*, *BRAF*, *PTEN* or *PIK3CA*. Similar results were obtained with PDOs, with *KRAS*^G12^-mutated lines consistently showing reduced FTD responsiveness (two-sided Wilcoxon rank-sum-based *P* = 0.034; Fig. 4e,f). Notably, the presence of a *KRAS*^G12^ mutation was associated with suppression of FTD-induced DNA damage (as measured by γH2AX) in both isogenic cell lines and PDOs (Fig. 4g,h). We next tested in vitro sensitivity to 5-FU because this chemotherapeutic is closely related to FTD/TPI but exerts its main effect through damaging RNA rather than DNA. In all models, *KRAS*^G12^ mutations did not significantly reduce in vitro sensitivity to 5-FU (Fig. 4i–l). Of note, the higher sensitivity to FTD in *KRAS*^WT^ models could not be explained by higher baseline proliferation rates, as the (untreated) *KRAS*^WT^ PDOs demonstrated lower proliferation rates than (untreated) *KRAS*^G12^ PDOs (Extended Data Fig. 8). Taken together, these results show that *KRAS*^G12^ mutation-based resistance to FTD can be modeled in vitro and is characterized by limited FTD-induced DNA damage.

## Discussion

Using two independent real-world datasets from three different countries and an independent validation cohort based on the global, double-blind, placebo-controlled, phase 3 RECOURSE trial, we demonstrate that codon-specific *KRAS* mutations predict OS benefit for patients treated with the chemotherapeutic agent FTD/TPI in late-stage mCRC. Specifically, *KRAS*^G12^ mutations identify patients who experience no clinically relevant^18^ survival benefit from FTD/TPI, while the remaining population—including *KRAS*^G13^-mutated patients—benefits substantially. The RECOURSE trial showed only a modest OS benefit of FTD/TPI versus placebo in the general, unselected population with mCRC. In this context, our results offer a framework to (re)assess the risk–benefit profile of FTD/TPI according to codon-specific *KRAS* mutations. Given that *KRAS* testing is routinely performed in the molecular workup of all patients with CRC to guide treatment with EGFR-targeting agents^1,11^, our findings can be readily adopted in the clinic.

In line with previous clinical and preclinical evidence^14–16^, our data demonstrate that *KRAS*^G12^ and *KRAS*^G13^ mutated mCRC are different clinical entities. The former disease is characterized by better prognosis but shows no clinically relevant OS benefit of FTD/TPI treatment (predictive effect), whereas the latter disease behaves aggressively when treated with placebo but can be more effectively managed with FTD/TPI treatment. These data caution against lumping together *KRAS* mutations at different codons in biomarker analyses and clinical trial designs because different biological and biochemical properties may be associated with different clinical outcomes.

The primary objective of the RECOURSE trial was to detect differences in OS between FTD/TPI and placebo, the gold standard outcome measure for regulatory approval studies for new treatments for metastatic cancer^19,20^. One of the reasons for this is that marginal improvements in PFS may not translate into an OS benefit^21^ as we observe in the subpopulation of the RECOURSE trial with *KRAS*^G12^ mutant tumors. The main caveat of OS is that lines of treatment administered after progression on the study drug might bias the conclusions. Notably, information on 5-FU-based rechallenges was not collected in the RECOURSE trial but are unlikely to underlie the reduced OS benefit of FTD/TPI in the population with *KRAS*^G12^ mutations. The reason is that this would require that placebo-treated patients with *KRAS*^G12^ mutations received considerably more treatments after progression in the study than FTD/TPI-treated patients with *KRAS*^G12^ mutations. Nevertheless, even in this unlikely scenario the conclusion would still be that, in terms of OS, the treatment with FTD/TPI has not been a useful intervention because it did not provide a relevant OS benefit over treatment with placebo.

Analysis of the real-world validation cohort showed that mismatch repair (MMR) deficiency was rare (Table 1) and was not associated with *KRAS*^G12^ status nor with OS of patients treated with FTD/TPI (data not shown). Furthermore, tumor sidedness was well balanced among all *RAS*/*RAF*-based subgroups of the real-world cohort and adjustment for this covariate in multivariate models did not affect our conclusions. In addition, pretreatment variables, such as the number of previous regimens, refractoriness to fluoropyrimidine or previous use of regorafenib were not responsible for our results. Namely, our RECOURSE trial-based analyses showed that these (1) were well balanced between the populations with *KRAS*^G12^ and *KRAS*^G13^ mutant tumors, (2) were not associated with OS benefit of FTD/TPI versus placebo and (3) did not alter our conclusions when incorporated into multivariate models.

While all *RAS*/*RAF*-based subgroups were molecularly well defined in our real-world datasets, this classification was not as complete in the RECOURSE trial. Indeed, *KRAS* hotspot mutations outside codons G12 and G13 were only tested in a small fraction of the RECOURSE trial population and data on *NRAS* and *BRAF* mutations were (largely) missing. Given the results of our real-world analyses, patients with *KRAS* mutations outside of codons G12 and G13 or *BRAF* mutations may more closely resemble patients with *KRAS*^G12^ mutations; inclusions of these cases in the *KRAS*^WT^ group might have underestimated the survival benefit conferred by FTD/TPI in the ‘real’ *KRAS*^WT^ population. A further observation with potential clinical implications relates to the fact that virtually all *KRAS*^WT^ patients in our cohorts were pretreated with anti-EGFR therapeutics, while their *RAS* (and *RAF*) status was determined before any therapy. Given that *RAS* mutations can emerge as drivers of acquired resistance in this scenario^22^, some patients might have been misclassified. Although the above considerations are important to keep in mind when interpreting the results, our conclusions hold regardless because such misclassifications may only have diluted the differences between the analyzed subgroups.

A potential limitation of our study is that this investigator-initiated reanalysis of the RECOURSE trial was not predefined in the original trial protocol. However, based on our findings, this reanalysis was hypothesis-driven and prespecified in a formal data request before access to the RECOURSE trial data was granted.

Several clinico-pathological and molecular biomarkers of benefit to FTD/TPI have been tested but none has reached clinical application^23^. Our results show that *KRAS* mutational analysis, a standard-of-care test already implemented worldwide, can identify patients with *KRAS*^G12^ mutant mCRC who are unlikely to benefit from FTD/TPI treatment, avoiding unnecessary toxicities to patients and rationalizing the use of resources for healthcare systems. Thus, we report the first proof of genomics-based precision medicine for a chemotherapy in mCRC, which has the potential to substantially improve patient selection for FTD/TPI treatment.

## Methods

### Study participants

#### Discovery cohort

The large, publicly available, real-world dataset with clinical annotation and whole-genome sequencing (WGS) by the HMF was used as the discovery cohort^17^. All patients who received FTD/TPI as part of their standard-of-care treatment for mCRC were identified in May 2018 (Supplementary Table 2). These patients were included in 13 academic, teaching, and regional hospitals in the Netherlands. The study was approved by the Medical Ethical Committee of the University Medical Center Utrecht and was conducted in accordance with the Declaration of Helsinki (fourth edition). All patients provided written informed consent for the collection, analysis and pseudonymized sharing of paired tumor-normal WGS data and clinical characteristics for research purposes.

#### Real-world validation cohort

For validation, we retrospectively collected data of 1,012 patients with mCRC treated with FTD/TPI as part of standard of care between April 2016 and January 2022 at 36 academic, teaching and regional hospitals across Italy and the UK (Supplementary Tables 6 and 7). The data cutoff was April 2022. Tumor *KRAS*, *NRAS* and *BRAF* genotype was investigated locally as recommended by local guidelines. Fifty-two patients with unknown *NRAS* or *BRAF* status were excluded, resulting in a final cohort of 960 patients used for all analyses. For patients from the UK, the study built on a UK National Audit^24^ and data were handled in accordance with the Declaration of Helsinki. Formal ethical approval for data collection, analysis and pseudonymized sharing for research purposes was covered by UK Health Research Authority guidance (NHS Health Research Authority, Service Evaluation Clinical/Non-Financial Audit Usual Practice (in Public Health Including Health Protection)). For patients from Italy, data collection, analysis and pseudonymized sharing for research purposes was approved by the institutional review board of the Fondazione Istituto di Ricovero e Cura a Carattere Scientifico Ca’ Granda Ospedale Maggiore Policlinico (Milano, Italy) and was conducted in accordance with the Declaration of Helsinki.

#### RECOURSE trial cohort

The RECOURSE trial design has been previously described in detail^3^. Briefly, the RECOURSE trial (NCT01607957) was an international double-blind, randomized, placebo-controlled, phase 3 trial comparing FTD/TPI plus best supportive care to placebo plus best supportive care. Heavily pretreated patients with refractory mCRC (*n* = 800) were randomly assigned in a 2:1 ratio to receive FTD/TPI or placebo. Within this process, patients were stratified based on *KRAS* status (mutant yes/no), time between first diagnosis of metastases and randomization (<18 versus ≥18 months) and geographical region (Japan or USA, Europe and Australia). The data cutoff was at 571 deaths, in accordance with the cutoff of the primary analysis. All patients in the study provided written informed consent, as stated in the original publication^3^.

#### Memorial Sloan Kettering Cancer Center CRC cohort

Somatic mutation data were downloaded from the cBioPortal for cancer genomics (http://cbioportal.org/msk-impact) on 8 August 2017. All samples with ‘GeneralTumorType’ = ‘Colorectal Cancer’ were included (Supplementary Table 1).

#### PDO cohort

PDOs were cultured from tumor biopsies of patients with mCRC, with approval of the Medical Ethical Committee of the Netherlands Cancer Institute. We used four *KRAS*^G12^-mutated (SNS26: *KRAS*^G12S^; TUM10: *KRAS*^G12V^; TUM3: *KRAS*^G12A^; TUM52: *KRAS*^G12C^) and three *KRAS*^WT^ (TUM42, TUM50, TUM65) PDOs (Supplementary Table 9). The study was conducted in accordance with the Declaration of Helsinki. All patients provided written informed consent for organoid culture and collection, analysis and pseudonymized sharing of clinical characteristics for research purposes.

### End points and study objectives

In the real-world discovery analysis, we searched for genome-wide somatic variants associated with OS and time on FTD/TPI treatment as end points. In the real-world validation analysis, the primary and secondary objectives were to assess the association of *KRAS*^G12^ mutation status with OS and PFS, respectively, both in the population as a whole and in *RAS*/*RAF* mutation-based subpopulations. All end points used in the real-life analyses were measured from the start of FTD/TPI treatment and evaluated at participating institutions over the treatment course according to local practice. In our reanalysis of the RECOURSE trial, we tested OS benefit and PFS benefit of FTD/TPI versus placebo as the primary and secondary end points, respectively, in subgroups defined by codon-specific *KRAS* mutation status. This was in accordance with the hierarchy of end points prespecified in the RECOURSE trial protocol; these reanalyses were prespecified in a formal data request to the sponsor of the RECOURSE study before access to the data was granted.

### Bioinformatics analysis

All genomics data of the discovery cohort was publicly available and provided by the HMF under the approved data request DR-015. WGS (median depths approximately 100 and approximately 40 for tumor and normal, respectively) and bioinformatics analysis of the discovery cohort were performed by the HMF as described previously^17^, with an optimized pipeline based on open-source tools freely available on GitHub (https://github.com/hartwigmedical/pipeline5). Somatic genomic drivers were identified as an integrated functionality of PURPLE v.2.43 (ref. ^17^). Briefly, somatic mutations were considered drivers if they fulfilled one of the following criteria: (1) mutations in oncogenes located at—or within five bases of—known hotspots; (2) inframe indels in oncogenes with repeat count <8 repeats; (3) biallelic (that is, the WT allele is lost) nonsense, splice or indel variants in tumor suppressor genes (TSGs); and (4) mutations in oncogenes or TSGs with a sample-specific driver likelihood >80%, as calculated by PURPLE as described previously^17^. For this manuscript, we only considered TSG mutations to be drivers if (1) they were biallelic or (2) in the case of multiple mutations in the gene for which the summed variant ploidies exceeded the gene ploidy within the sample −0.5 (for example, the classical APC two-hit hypothesis). Amplifications were considered to be drivers if (1) they affected an oncogene with pan-cancer evidence for recurrent amplification^17^ and (2) this oncogene had a copy number exceeding three times the sample ploidy. Deletions were considered to be drivers if (1) they affected TSGs with pan-cancer evidence for recurrent deletion^17^ and (2) they were homozygous (absolute gene copy number <0.5).

### Statistical analysis

Median time on treatment, OS and PFS were calculated using the Kaplan–Meier method. OS and time on treatment were compared between biomarker positive versus negative patients in the discovery cohort using the exact log-rank test. In this analysis, multiple-hypothesis correction was performed using the Benjamini–Hochberg procedure. HRs and corresponding 95% CIs, and Wald test-based two-sided *P* values, were estimated from Cox regression models. The proportional-hazards assumption was tested using the methodology developed by Grambsch and Therneau^25^, with a significance threshold of *P* = 0.05; categorical covariates were modeled as stratification factors (rather than covariates) where appropriate to prevent assumption violations. ‘Unadjusted’ Cox regression analyses of the real-world validation cohort were performed in a univariate manner. ‘Adjusted’ Cox regression analyses of the real-world validation cohort were stratified for ECOG performance status (0–1 versus ≥2) and adjusted for seven additional covariates: time since diagnosis of first metastases (<18 versus ≥18 months); geographical region (UK versus Italy); age (<65 versus ≥65 years); sex; sidedness (left versus right); previous surgery (yes versus no); and peritoneal disease at the start of FTD/TPI treatment (yes versus no). For the RECOURSE trial-based analyses, ‘unadjusted’ Cox regression was stratified for two stratification factors of the trial: time since diagnosis of first metastases (<18 versus ≥18 months) and geographical region (Japan versus the USA, Europe and Australia). The third stratification factor of the trial, *KRAS* mutation status, was omitted because of high collinearity with our variables of interest (codon-specific *KRAS* status). For ‘adjusted’ RECOURSE trial-based analyses, we used stratified, multivariate Cox regression to adjust for eight prognostic factors on top of the two stratification factors used in the unadjusted analyses. These included: age (<65 versus ≥65 years); sex; ECOG performance status (0 versus 1); primary site of the disease (colon versus rectum); disease refractory to fluoropyrimidine as part of the last previous regimen (yes versus no); previous use of regorafenib; number of previous regimens (2, 3 or ≥4); and number of metastatic sites (1–2 versus ≥3). The rationale behind the selection of covariates for multivariate Cox regression is specified below. No subsequent covariate selection was performed; hence, all eight covariates plus the two stratification factors were included in all multivariate models of RECOURSE trial data. None of these variables were predictive of FTD/TPI benefit in the RECOURSE trial (*P* > 0.20 for all variables)^3^. Dose–response curves were fitted using Prism v.9.0.0 (GraphPad Software) on log_2_-transformed FTD concentration values versus viability; the resulting fitted curves were then used to calculate IC_50_ values. Baseline characteristics were compared by Fisher’s exact test for categorical variables with two levels and by chi-squared test in the case of more than two levels. All reported *P* values are two-sided. In the main text and figures, all *P* values smaller than 0.001 were reported as <0.001. RECOURSE trial data-based survival analyses were performed on the intention-to-treat population and were prespecified in a formal data request before access to the data was granted.

### Candidate biomarker selection for the discovery cohort

The procedure for the selection of candidate biomarkers was as follows. Somatic genomic driver alterations (mutations and copy number alterations) were included as candidate biomarkers at increasingly specific ‘levels’: (1) gene-level biomarkers, for example, ‘*APC* alteration’, which could either be by mutation or copy number alteration; (2) variant class-level biomarkers, for example, ‘*APC* mutation’ or ‘*APC* deletion’; (3) codon-level biomarkers, for example, ‘*APC* codon 1450 mutation’; and (4) amino acid change-specific biomarkers, for example, ‘*APC* p.Thr562Met mutation’. In cases where biomarkers of different levels showed complete redundancy, only the most specific level was included. For example, all *KRAS* alterations in the cohort were mutations leading to complete redundancy between ‘*KRAS* alteration’ and ‘*KRAS* mutation’. Hence, *KRAS* mutation was selected as the most specific level and included as a candidate biomarker, whereas *KRAS* alteration was excluded. All candidate biomarkers occurring in at least five patients in the discovery cohort were tested for association with treatment outcomes. Supplementary Table 3 provides a comprehensive overview of the frequencies of all candidate biomarkers identified in our cohort.

### Variable selection for multivariate Cox regression

#### Real-world validation cohort

We selected eight variables for multivariate (adjusted) Cox proportional-hazards modeling of the real-world validation cohort. In this process, we aimed to harmonize the selection as much as possible to the variables used in the Cox regression modeling of the RECOURSE trial-based data (see below), with some alterations.

The ECOG performance status (0–1 versus ≥2) was used as a stratification factor because this variable violated the proportional-hazards assumption when modeled as a covariate. Furthermore, we adjusted for seven additional covariates: time since diagnosis of first metastases (<18 versus ≥18 months); geographical region (UK versus Italy); age (<65 versus ≥65 years); sex; sidedness (left versus right); previous surgery (yes versus no); and peritoneal disease at the start of FTD/TPI treatment (yes versus no). Sidedness was used instead of primary site of the disease (colon versus rectum, as used in the RECOURSE trial-based analyses), because sidedness was most strongly associated with OS and only one of these two variables could be included due to high collinearity. Due to data unavailability for the real-world validation cohort, we were unable to factor in if the disease was refractory to fluoropyrimidine as part of the last previous regimen, previous use of regorafenib, the number of previous regimens and the number of metastatic sites in the analyses. In RECOURSE trial-based analyses, none of these factors were predictive and only the latter variable was prognostic for OS. Instead, based on significant (univariate) associations with OS in the real-world validation cohort, we decided to add the two variables ‘previous surgery’ and ‘peritoneal disease at the start of FTD/TPI treatment’ to our selection of covariates, although these data were unavailable for the RECOURSE trial dataset.

#### RECOURSE trial-based analyses

We selected ten variables for multivariate (adjusted) Cox proportional-hazards modeling of RECOURSE trial data.

This selection included all factors prespecified in the RECOURSE trial study protocol, except *KRAS* status and ethnicity, totaling eight prespecified factors: time since diagnosis of first metastases (<18 versus ≥18 months (stratification factor of the study); geographical region (Japan versus the USA, Europe and Australia; stratification factor of the study); age (<65 versus ≥65 years); sex; ECOG performance status (0 versus 1); primary site of the disease (colon versus rectum); number of previous regimens (2, 3 or ≥4); and number of metastatic sites (1–2 versus ≥3). *KRAS* status was excluded because of collinearity with our variables of interest (*KRAS*^G12^ mutation, *KRAS*^G13^ mutation, *KRAS*^WT^). Ethnicity was excluded for two reasons. First, the sponsor of the RECOURSE trial could not share the original ethnicity data for privacy reasons because the number of Black participants (nine patients) was below a predefined threshold put in place to prevent patient reidentification. For this reason, the ethnicity item has been modified to a quasi-identifier of ‘Asian’ versus ‘Other’ (White or Black). In the RECOURSE trial, the original ethnicity variable was not significantly prognostic or predictive for OS^3^. Second, the modified ethnicity variable showed high collinearity (and hence redundancy) with the included factor ‘geographical region’ because 266 out of 266 (100%) of participants from the ‘Asia’ region had the ‘Asian’ ethnicity and 522 out of 534 (98%) of participants from the USA, Europe and Australia regions had the ‘Other’ (which included Black and White) ethnicity.

Next, we included two additional factors in our multivariate models: (1) disease refractory to fluoropyrimidine as part of the last previous regimen; and (2) previous use of regorafenib. These factors were not prespecified in the RECOURSE trial protocol for multivariate analyses but were used for the subgroup analyses reported by Mayer et al.^3^. We decided to include these pretreatment-related factors in our multivariate models because patients with *KRAS* mutant tumors showed significant differences regarding their pretreatment profiles as compared to patients with *KRAS*^WT^ tumors. Patients with *KRAS* mutant tumors were more often refractory to fluoropyrimidine as part of their last previous regimen and were less heavily pretreated than patients with *KRAS*^WT^ (Table 2).

*BRAF* mutation status was not included in our selection because this information was missing for 676 out of 800 (85%) patients. Subgroup analysis of the population with *BRAF* mutant tumors was not possible because *BRAF* mutations were detected in only eight patients.

### Organoid and cell line cultures and drug assays

PDOs were cultured, expanded and assayed as described previously^22^. FTD (catalog no. S1778, Selleckchem) and 5-FU (catalog no. S1209, Selleckchem) were reconstituted in DMSO (catalog no. D2650, Sigma-Aldrich) at a stock concentration of 50 mM. PDOs were exposed to a two-step, eightfold dilution of FTD (range = 0.781–200 μM) for 11 d or to 5-FU for 6 d in a two-step, eightfold dilution (range = 0.781–200 μM). Culture medium and FTD were refreshed every 3–4 d. The isogenic cell lines were assayed with FTD and 5-FU in a similar fashion, shortening the assay duration to 3 d. The used concentrations were adjusted to include more data points of lower concentrations (range = 0.1 nM, 0.5 nM, 1 μM, 2 μM, 5 μM, 10 μM, 20 μM, 50 μM, 200 μM, 500 μM). The readout was performed using the MTT Assay Kit for Cell Proliferation (catalog no. ab211091, Abcam); culture medium was replaced with 100 μl of a 1:1 mix of MTT reagent with serum-free RPMI 1640 (catalog no. 21875034, Gibco), which was replaced by 150 μl MTT solvent after incubation, according to the manufacturer’s protocol. Then, absorbance was measured at OD_590 __nM_ on an Infinite 200 Pro plate reader (Tecan Life Sciences).

### Isogenic cell line construction: Colo320 *KRAS*^G12D^ knock in

Single-guide RNA oligonucleotide sequences were designed on Chop-Chop (http://chopchop.cbu.uib.no/#). CRISPR–Cas9 CRISPR RNA (crRNA) (5′-CUUGUGGUAGUUGGAGCUGG-3′) and trans-activating crRNA (tracrRNA) (catalog no. 1072532), Cas9 Nuclease V3 (catalog no. 1081058) and HDR Donor Oligo (5′-ATTCTGAATTAGCTGTATCGTCAAGGCACTCTTGCCTACGCCGTCAGCTCCCACTACCACAAGTTTATATTCAGTCATTTTCAGC-3′) were purchased from Integrated DNA Technologies. Briefly, guide RNA (gRNA) complexes were formed as described previously^26^ by combining equal amounts of crRNA (160 μM in stock) and tracrRNA (160 μM in stock) in Duplex Buffer (cat no. 11-01-03-01, Integrated DNA Technologies) and heating the oligonucleotides to 95 °C, followed by slowly cooling to room temperature. Cas9 nuclease was then added (the molar ratio of crRNA:Cas9 nuclease was 1:0.5) to the gRNA complexes, followed by 15-min incubation at room temperature. The Cas9 ribonucleoprotein (ctRNP) complexes were then stored on ice until use. DNA HDR templates were prepared by diluting the HDR Donor Oligo stock to 10 μM in nuclease-free water. Electroporation was performed by using the 4D-Nucleofector X Unit (catalog no. AAF-1003X, Lonza) according to the manufacturer’s instructions. For each sample, 2 × 10^5^ cells were resuspended in Ingenio Electroporation Solution (catalog no. MIR 50111, Mirus Bio). Per reaction, 2.5 μM ctRNP and 0.5 μM HDR template were added to the cell suspension. We next pipetted 20 μl of each sample into individual wells of 16-well Nucleocuvette Strips (catalog no. AXP-1004, Lonza) and ran the program CM-137. After electroporation, cells were sorted by FACS and single cells were cultured in 96-well plates for up to two weeks. For single-cell clones, the presence of the *KRAS*^G12D^ mutation was then confirmed by Sanger sequencing.

### Western blot analysis

Western blot analysis was performed on the isogenic cell lines and PDOs treated with FTD or 5-FU, at different concentrations, for 24 h (cell lines) or 48 h (PDOs). For PDOs (but not for the isogenic cell lines), the extracellular matrix was removed by incubating with 2 mg ml^−1^ type II dispase (catalog no. D4693, Sigma-Aldrich) for 10 min at 37 °C. Cells were washed with PBS and lysed in RIPA Lysis and Extraction Buffer (catalog no. 89901, Thermo Fisher Scientific), supplemented with Phosphatase Inhibitor Cocktail (catalog no. 78420, Thermo Fisher Scientific) and Halt Protease Inhibitor Cocktail (catalog no. 87786, Thermo Fisher Scientific). Protein concentration was determined using Coomassie Brilliant Blue G-250 (catalog no. 1610803, Bio-Rad Laboratories). Protein samples were run on NuPAGE 4–12% Bis-Tris Gels (catalog no. NP0323BOX, Thermo Fisher Scientific), transfer was performed using the iBlot 2 Gel Transfer Device (catalog no. IB21001, Thermo Fisher Scientific) and compatible iBlot Transfer Stack; nitrocellulose (catalog no. IB301002, Thermo Fisher Scientific) membranes were used. Membranes were blocked in 5% BSA (catalog no. 10735094001, Sigma-Aldrich) in PBS plus 0.2% Tween-20 (catalog no. P1379-1L, Sigma-Aldrich) for 1 h, then incubated with primary antibodies in 5% BSA in PBS and Tween-20. As primary antibodies, we used anti-phospho-Histone H2A.X (Ser139, catalog no. 05-636, Sigma-Aldrich) and anti-HSP 90α/β (catalog no. sc-13119, Santa Cruz Biotechnology), which were diluted 1:1,000 in PBS with 5% BSA. The secondary antibody (anti-mouse IgG, HRP-linked antibody, catalog no. 7076, Cell Signaling Technology) was diluted 1:1,000 in 5% BSA in PBS and Tween-20. The blots were incubated with Clarity Max Western ECL Substrate (catalog no. 1705062, Bio-Rad Laboratories) and the luminescence signal was imaged using the ChemiDoc Imaging System (catalog no. 17001401, Bio-Rad Laboratories).

### Colony formation assay

Cells were seeded into six-well plates (1.5–2 × 10^4^ cells per well) and cultured in the presence of drugs at the indicated concentrations. For each cell line, cells cultured using different conditions were fixed in methanol (catalog no. 32213, Honeywell) and stained with 0.1% crystal violet solution (catalog no. V5265, Sigma-Aldrich).

### Role of the funding source

The funders of the study had no role in study design, data collection, data analysis, data interpretation or writing of the article. Authors had full access to all the data and had the final responsibility to submit for publication.

### Statistical methods and associated software

The statistical methods and associated packages used in this study are summarized in Table 3.

**Table 3:** Statistical methods and associated software

Statistical method	Programming language	Package	Version	Function
Kaplan–Meier method	R v.3.6.1	survminer	0.4.6	surv_median
Kaplan–Meier curve plotting	R v.3.6.1	survminer	0.4.6	ggsurvplot
Exact log-rank test	R v.3.6.1	coin	1.3.1	logrank_test (distribution = ‘exact’)
Cox regression	R v.3.6.1	survival	3.2–7	coxph
Proportional-hazards assumption testing	R v.3.6.1	survival	3.2–7	cox.zph
Wilcoxon rank-sum test	Python 3	scipy	1.4.1	stats.ranksums
Fisher’s exact test	Python 3	scipy	1.4.1	stats.fisher_exact
Chi-squared test	Python 3	scipy	1.4.1	stats.chi2_contingency

### Reporting summary

Further information on research design is available in the Nature Portfolio Reporting Summary linked to this article.

## Online content

Any methods, additional references, Nature Portfolio reporting summaries, source data, extended data, supplementary information, acknowledgements, peer review information; details of author contributions and competing interests; and statements of data and code availability are available at 10.1038/s41591-023-02240-8.

## Supplementary information

Reporting Summary

Supplementary Tables Supplementary Tables 1–9.

Source Data Fig. 4. Source data for Fig. 4g,h.

## Data Availability

The somatic mutation data of the MSKCC cohort are freely available via the cBioPortal for cancer genomics (http://cbioportal.org/msk-impact); patient identifiers are provided in Supplementary Table 1. The sequencing data of the HMF cohort (discovery cohort) can be accessed through the Hartwig Medical Foundation upon approval of a research access request (https://www.hartwigmedicalfoundation.nl/en/data/data-acces-request). The patient identifiers, patient-level clinical outcome data and biomarker status of all patients in this cohort are provided in Supplementary Table 2. The original data used in all analyses of the real-world validation cohort can be found in Supplementary Table 7. The RECOURSE trial data can be accessed upon approval of a data request at Servier (https://clinicaltrials.servier.com/data-request-portal/). Source data are provided with this paper.

## Code availability

The bioinformatics for the WGS data of the discovery cohort were performed with an optimized pipeline based on open-source tools; this is freely available on GitHub (https://github.com/hartwigmedical/pipeline5).
