# Supplementary material for: Codon-specific KRAS mutations predict survival benefit of trifluridine/tipiracil in metastatic colorectal cancer
Source: Nat Med. 2023 Mar 2;29(3):605–14. doi: 10.1038/s41591-023-02240-8 (PMC10033412; doi:10.1038/s41591-023-02240-8)
Supplement: Supplementary file 1 — Reporting Summary [file 41591_2023_2240_MOESM1_ESM.pdf]

nature portfolio

Corresponding author(s): Emile E. Voest, Nicola Valeri

Last updated by author(s): Jan 12, 2023

Reporting Summary

Nature Portfolio wishes to improve the reproducibility of the work that we publish. This form provides structure for consistency and transparency in reporting. For further information on Nature Portfolio policies, see our Editorial Policies and the Editorial Policy Checklist.

Statistics

For all statistical analyses, confirm that the following items are present in the figure legend, table legend, main text, or Methods section.

n/a	Confirmed	
☐	☒	The exact sample size (*n*) for each experimental group/condition, given as a discrete number and unit of measurement
☐	☒	A statement on whether measurements were taken from distinct samples or whether the same sample was measured repeatedly
☐	☒	The statistical test(s) used AND whether they are one- or two-sided<br>*Only common tests should be described solely by name; describe more complex techniques in the Methods section.*
☐	☒	A description of all covariates tested
☐	☒	A description of any assumptions or corrections, such as tests of normality and adjustment for multiple comparisons
☐	☒	A full description of the statistical parameters including central tendency (e.g. means) or other basic estimates (e.g. regression coefficient) AND variation (e.g. standard deviation) or associated estimates of uncertainty (e.g. confidence intervals)
☐	☒	For null hypothesis testing, the test statistic (e.g. *F*, *t*, *r*) with confidence intervals, effect sizes, degrees of freedom and *P* value noted<br>*Give P values as exact values whenever suitable.*
☒	☐	For Bayesian analysis, information on the choice of priors and Markov chain Monte Carlo settings
☒	☐	For hierarchical and complex designs, identification of the appropriate level for tests and full reporting of outcomes
☐	☒	Estimates of effect sizes (e.g. Cohen's *d*, Pearson's *r*), indicating how they were calculated

Our web collection on statistics for biologists contains articles on many of the points above.

Software and code

Policy information about availability of computer code

Data collection	Bioinformatics for whole genome sequencing data were performed as previously described, with an optimized pipeline based on open source tools, including PURPLE v2.43 for somatic driver calling, which is freely available on GitHub (https://github.com/hartwigmedical/pipeline5). For other data, did not use specific computer code for collection.
Data analysis	Kaplan-Meier method: R package = survminer 0.4.6; function = surv_median<br>Kaplan-Meier curve plotting: R package = survminer 0.4.6; function = ggsurvplot<br>Cox regression: R package = survival 3.2.7; function = coxph<br>Cox proportional hazards assumption testing: R package = survival 3.2.7; function = cox.zph<br>Fisher's exact test: Python 3 package = scipy 1.4.1; function = stats.fisher_exact<br>Chi2 test: Python 3 package = scipy 1.4.1; function = stats.chi2_contingency<br>Wilcoxon rank sum test = scipy 1.4.1; function = stats.ranksums<br>Dose-response curve fitting: Graphpad Prism 9.0.0

For manuscripts utilizing custom algorithms or software that are central to the research but not yet described in published literature, software must be made available to editors and reviewers. We strongly encourage code deposition in a community repository (e.g. GitHub). See the Nature Portfolio guidelines for submitting code & software for further information.

Data

Policy information about availability of data

All manuscripts must include a data availability statement. This statement should provide the following information, where applicable:

- Accession codes, unique identifiers, or web links for publicly available datasets
- A description of any restrictions on data availability
- For clinical datasets or third party data, please ensure that the statement adheres to our policy

Discovery cohort HMF
Sample identifiers and patient-level biomarker and clinical outcome data are available in Supplementary Table 2. Raw and processed genomics data are freely available at Hartwig Medical Foundation through standardized procedures and request forms (https://www.hartwigmedicalfoundation.nl/en/data/data-acces-request).

Real-World validation cohort
The original data used in all analyses of the real-world validation cohort can be found Supplementary Table 7.

RECOURSE trial
The RECOURSE trial data can be accessed upon approval of a data request at Servier (https://clinicaltrials.servier.com/data-request-portal/).

MSKCC Colorectal Cancer Cohort
Somatic mutation data of the MSKCC cohort are freely available via the cBioportal for cancer genomics (http://cbioportal.org/msk-impact) and patient identifiers are provided in Supplementary Table 1.

Human research participants

Policy information about studies involving human research participants and Sex and Gender in Research.

Reporting on sex and gender

Throughout the manuscript, we report sex as an biological attribute, which was collected through self reporting. Sharing of patient-level, pseudonymized clinical data, including sex, for research purposes was covered by the informed consents of the individual studies and local legislation.

Population characteristics

Discovery cohort HMF
Patients with advanced or metastatic cancer for whom there is an indication for any systemic treatment were included in the HMF database as part of the CPCT-02 (NCT01855477) clinical study. We included all patients in the HMF database who received FTD/TPI as part of their standard-of-care treatment for advanced/metastatic, histologically proven, colorectal carcinoma, which were identified in the HMF database in May 2018. The median age was 62 years (IQR 59-68), 38% was (self-reported) female and 62% male, and the percentage of patients with KRAS G12 and other KRAS mutations were 54% and 11%, respectively. All patients received FTD/TPI treatment as part of standard-of-care. As FTD/TPI is approved as final line therapy, these were all late-stage patients.

Real-World Cohort (UK and Italy)
Retrospective collection of all sequential patients with advanced/metastatic, histologically proven, colorectal carcinoma undergoing treatment with FTD/TPI following progression to prior lines of standard chemotherapy, with known codon-specific RAS/RAF status known between November 2016 and March 2022. As reported in Table 1, the median age was 64 years (IQR 56-71), 41% was (self-reported) female and 59% male, 72% and 28% were diagnosed with colon and rectum cancer, respectively, and the percentage of patients with KRAS G12, KRAS G13, KRAS other, NRAS and BRAF mutations were 36%, 9.0%, 5.5%, 6.4%, and 3.3%, respectively. All patients received FTD/TPI treatment as part of standard-of-care. As FTD/TPI is approved as final line therapy, these were all late-stage patients.

RECOURSE trial (population description from the original publication, by Mayer et al, NEJM 2015)
"Baseline demographic and disease characteristics were well balanced between the two study groups (Table 1). All the patients had received prior chemotherapy regimens containing a fluoropyrimidine, oxaliplatin, and irinotecan; all but one patient (in the placebo group) had received bevacizumab. All but two patients (one patient in each study group) with KRAS wild-type tumors had received cetuximab or panitumumab. Regorafenib, an oral multikinase inhibitor, became available for the management of previously treated colorectal cancer during the course of the study; 17% of the patients in the TAS-102 group, as compared with 20% of those in the placebo group, had received this drug. A large percentage of patients in both study groups — 93% of patients receiving TAS-102 and 90% of those receiving placebo — had disease that had been refractory to fluoropyrimidines when they were last exposed to this class of drugs. Moreover, 58% of the patients receiving TAS-102 and 54% of the patients receiving placebo had disease that had been refractory to fluoropyrimidine when that drug was administered as part of their last treatment regimen before study entry."

"Patients with biopsy-documented adenocarcinoma of the colon or rectum were eligible for participation in the study if they had received at least two prior regimens of standard chemotherapies, which could have included adjuvant chemotherapy if a tumor had recurred within 6 months after the last administration of this therapy; if they had either tumor progression within 3 months after the last administration of chemotherapy; or if they had had clinically significant adverse events from standard chemotherapies that precluded the readministration of those therapies. Eligibility also required knowledge of tumor status with regard to KRAS (i.e., wild-type or mutant), as reported by investigators. Patients were also required to have received chemotherapy with each of the following agents: a fluoropyrimidine, oxaliplatin, irinotecan, bevacizumab, and — for patients with KRAS wild-type tumors — cetuximab or panitumumab. In addition, patients had to be 18 years of age or older; have adequate bone-marrow, liver, and renal function; and have an Eastern Cooperative Oncology Group (ECOG) performance

status of 0 or 1 (on a scale of 0 to 5, with 0 indicating no symptoms, 1 indicating mild symptoms, and higher numbers indicating increasing degrees of disability)."

Recruitment

Discovery cohort HMF
Patients with advanced or metastatic cancer for whom there is an indication for any systemic treatment were included in the HMF database as part of the CPCT-02 (NCT01855477) clinical study. The CPCT-02 study, patients were included by 41 academic, teaching and general hospitals across The Netherlands and collected material and clinical data by standardized protocols. Metastatic cancer patients were asked to participate in the studies in any of the 41 participating hospitals. Recruitment involved hundreds of medical specialists and research nurses which minimizes self-selection biases. Recruitment was independent on tumor type. An important requirement for participation was the ability to safely undergo a tumor biopsy. Health conditions and lesion site related risk could therefore have resulted in exclusion of patients.

Real-world validation cohort
As per above

RECOURSE trial (recruitment details from the original publication, by Mayer et al, NEJM 2015)
"Between June 17, 2012, and October 8, 2013, a total of 1002 patients were screened for eligibility, of whom 800 underwent randomization, with 534 assigned to receive TAS-102 and 266 assigned to receive placebo (intention-to-treat population) (details regarding the disposition of patients are provided in Fig. S1 in the Supplementary Appendix, available at NEJM.org). Treatment was initiated in 798 patients, with 533 receiving TAS-102 and 265 receiving placebo (safety-analysis population). All treated patients received their assigned study drug according to the randomization schema, and 760 could be evaluated for assessment of tumor response (tumor-response population)."

Ethics oversight

HMF discovery cohort
The study was approved by the Medical Ethical Committee of the University Medical Center Utrecht and was conducted in accordance with the Declaration of Helsinki. All patients provided written informed consent for collection, analysis and pseudonymized sharing paired tumor-normal whole genome sequencing data and clinical characteristics for research purposes.

Real-world validation cohort
For patients included from the UK, the study built on a UK National Audit (Stavraka et al. Clin Colorectal Cancer. 2021 Dec;20(4):342-349) and data were handled in accordance with the Declaration of Helsinki. Formal ethical approval for data collection, analysis and pseudonymized sharing for research purposes was covered by UK Health Research Authority guidance (NHS Health Research Authority. Service Evaluation Clinical/Non Financial Audit Usual Practice [in Public Health Including Health Protection]. London: NHRA). For patients included in Italy, data collection, analysis and pseudonymized sharing for research purposes was approved by the Institutional Review Board of the Fondazione IRCCS Ca' Granda Ospedale Maggiore Policlinico, Milano, Italy and was conducted in accordance with the Declaration of Helsinki.

RECOURSE trial (from the original publication, by Mayer et al, NEJM, 2015)
"The review board at each participating institution approved the study, which was conducted according to the principles of the Declaration of Helsinki and the International Conference on Harmonisation Good Clinical Practice guidelines. All patients provided written informed consent."

Patient-derived organoid (PDO) cohort
The study was approved by the Medical Ethical Committee of the Netherlands Cancer Institute and was conducted in accordance with the Declaration of Helsinki. All patients provided written informed consent for organoid culture and collection, analysis and pseudonymized sharing of clinical characteristics for research purposes.

Note that full information on the approval of the study protocol must also be provided in the manuscript.

Field-specific reporting

Please select the one below that is the best fit for your research. If you are not sure, read the appropriate sections before making your selection.

☒ Life sciences ☐ Behavioural & social sciences ☐ Ecological, evolutionary & environmental sciences

For a reference copy of the document with all sections, see nature.com/documents/nr-reporting-summary-flat.pdf

Life sciences study design

All studies must disclose on these points even when the disclosure is negative.

Sample size

A priori sample size calculations were not performed. Sample sizes were determined by the max amount of patients/samples with available data.

Discovery cohort: All patients in the HMF database who received FTD/TPI as part of their standard-of-care treatment for mCRC were identified in May 2018.

Real-World Cohort (UK and Italy): Clinical pathological and molecular data of all the sequential patient treated with FTD/TPI at 35 centers in Italy and UK between between November 2016 and March 2022 were retrieved accessing electronic patients records (n=1012). 52 patients were excluded from the analysis as explained below, leading the final number of analyzed cases to n=960.

RECOURSE trial: All 800 included patients.

MSKCC Colorectal Cancer Cohort: All samples with 'GeneralTumorType' = 'Colorectal Cancer' were included. TCGA Colorectal Cancer Cohort: All samples with 'Primary Site' = 'Colon' or 'Rectum' were included.

Data exclusions

Discovery cohort: No exclusions
RECOURSE trial: No exclusions
Real-World Cohort (UK and Italy): 52 patients were excluded as NRAS and/or BRAF status was not available.
MSKCC Colorectal Cancer Cohort: No exclusions
TCGA Colorectal Cancer Cohort: Only patients with available consensus molecular subtypes (CMS) were considered

Replication

The main finding of the manuscript that KRAS G12 mutations are associated with reduced OS in patients with mCRC upon treatment with FTD/TPI was found in a discovery cohort, replicated in a large real-world cohort and in the RECOURSE trial. The RECOURSE trial-based analysis showed that this was based on a predictive effect. This RECOURSE trial-based analysis was prespecified in a formal data request before access to the data was granted, which minimizes potential biases. The finding that KRAS G13 patients have improved OS upon treatment with FTD/TPI was discovered in the large real-world cohort, as these patients were absent in the smaller discovery cohort. This finding was then replicated with re-analysis of the RECOURSE trial (also pre-specified in our formal data request), which showed that this was based on a predictive effect.

In vitro results:
The colony formation assays (Figure 4A-B) were independently repeated three times with similar results.
FTD/TPI sensitivity testing of isogenic cells lines (Figure 4C-D) was independently repeated four times with similar results.
FTD/TPI sensitivity testing of KRAS wild type organoid lines (Figure 4E-F) was independently repeated three times with similar results.
FTD/TPI sensitivity testing of KRAS G12 mutant organoid lines (Figure 4E-F) was independently repeated four times with similar results.
Western blot analysis of SW48 isogenic cell lines (Figure 4G) was independently repeated three times with similar results.
Western blot analysis of Colo320 isogenic cell lines (Figure 4G) was independently repeated two times with similar results.
5-FU sensitivity testing of isogenic cells lines (Figure 4I-J) was independently repeated three times with similar results.
5-FU sensitivity testing of organoids (Figure 4K-L) was independently repeated three times with similar results.

Randomization

Experimental subgroups were either determined by KRAS codon-specific mutation status, a non-random procedure, or (in the RECOURSE trial) by treatment status (FTD/TPI vs placebo), a random procedure. In the real-world cohort-based analyses, we reported subgroup-specific differences in baseline characteristics in the main text and Table 1, and adjusted for these differences using multivariate Cox regression. In the RECOURSE trial-based analyses, we reported subgroup-specific differences in baseline characteristics in the main text and Table 2, and adjusted for these differences using multivariate Cox regression. Furthermore, we specifically compared the KRAS G12 mutant subgroup with the other RAS/RAF mutant subgroup, which were two subgroups with very similar baseline characteristics (see Table 1 and Table 2).

Blinding

Discovery cohort HMF
Collection of genomics data and bioinformatics analysis were performed prospectively and hence blinded for clinical outcomes. Collection of clinical outcomes was performed by trained research nurses and medical doctors at the sites of inclusion, who were blinded, as they lacked knowledge about the research question of our study. Data analysis happened in an unblinded fashion.

Real-world Cohort (UK and Italy)
Collection of clinical outcomes was performed by trained research nurses and medical doctors at the sites of inclusion, who were blinded, as they lacked knowledge about the research question of our study. Data analysis happened in an unblinded fashion.

RECOURSE trial
The RECOURSE trial was a double-blind study. Details on this process can be found in the original publication (Mayer et al, NEJM 2015). In this re-analysis, data analysis was prespecified in a formal and detailed data request before access to the data was granted, but happened in an unblinded fashion.

In vitro experimentation occurred in an unblinded fashion, as the experimenters generated the appropriate models for their experiments, but given standardized procedures for data readout (automated cell viability readouts and Western blotting) the risk of potential biases was limited.

Reporting for specific materials, systems and methods

We require information from authors about some types of materials, experimental systems and methods used in many studies. Here, indicate whether each material, system or method listed is relevant to your study. If you are not sure if a list item applies to your research, read the appropriate section before selecting a response.

Materials & experimental systems

n/a	Involved in the study
☐	☒ Antibodies
☐	☒ Eukaryotic cell lines
☒	☐ Palaeontology and archaeology
☒	☐ Animals and other organisms
☐	☒ Clinical data
☒	☐ Dual use research of concern

Methods

n/a	Involved in the study
☒	☐ ChIP-seq
☒	☐ Flow cytometry
☒	☐ MRI-based neuroimaging

Antibodies

Antibodies used	The following primary antibodies were used: phospho-Histone H2A.X (Ser139) (#05-636) was purchased from Sigma-Aldrich; HSP 90α/β (#sc-13119) was purchased from Santa Cruz Biotechnology.<br>The following secondary antibodies were used: Anti-mouse IgG, HRP-linked antibody (#7076, Cell Signaling Technology).<br>All antibodies were diluted 1:1000 in PBS plus 5% BSA.
Validation	phospho-Histone H2A.X (Ser139) (#05-636, Sigma-Aldrich): https://www.sigmaaldrich.com/NL/en/product/mm/05636i<br><br>HSP 90α/β (#sc-13119, Santa Cruz Biotechnology): https://www.scbt.com/p/hsp-90alpha-beta-antibody-f-8<br><br>Anti-mouse IgG, HRP-linked antibody (#7076, Cell Signaling Technology): https://www.cellsignal.com/products/secondary-antibodies/anti-mouse-igg-hrp-linked-antibody/7076

Eukaryotic cell lines

Policy information about cell lines and Sex and Gender in Research

Cell line source(s)	SW48 isogenic cell lines (KRAS wild type, KRAS G12V mutant): Provided by Alberto Bardelli (IFOM, Milan) and his team.<br>Colo320 parental cell line (KRAS wild type): Provided by Rene Bernards and his team.<br><br>All organoid lines were established within our own research group using patient-derived tumor material.
Authentication	SW48 isogenic cell lines: We confirmed KRAS WT/G12V status with targeted sequencing.<br>Colo320 isogenic cell lines: We confirmed KRAS WT/G12D status with targeted sequencing.<br>All organoid lines: we confirmed KRAS codon-specific mutation status with targeted sequencing and performed SNP testing on organoids and primary patient material to confirm the patient of origin.
Mycoplasma contamination	All cell and organoid lines were confirmed to be negative for Mycoplasma contamination.
Commonly misidentified lines<br>(See ICLAC register)	No commonly misidentified lines were used.

Clinical data

Policy information about clinical studies

All manuscripts should comply with the ICMJE guidelines for publication of clinical research and a completed CONSORT checklist must be included with all submissions.

Clinical trial registration	Discovery cohort HMF: NCT01855477<br>RECOURSE study: NCT01607957<br>Real-World Cohort (UK and Italy) retrospective analysis no clinical trial registration.
Study protocol	Discovery cohort HMF: The study protocol of NCT01855477 has not been published publicly, but is available upon reasonable request.<br>Real-World Cohort (UK and Italy) retrospective analysis. Study protocol available upon request.<br>RECOURSE study: https://www.nejm.org/doi/suppl/10.1056/NEJMoa1414325/suppl_file/nejmoa1414325_protocol.pdf
Data collection	Discovery cohort HMF<br>Data was collected by trained clinicians and research nurses at 13 academic, teaching, and regional hospitals throughout the Netherlands. All patients were accrued between April 2016 and January 2018.<br><br>Real-world validation cohort<br>Retrospective collection of clinical-pathological and molecular data was performed by trained clinicians and research nurses in 36 academic, teaching and regional hospitals in Italy and the UK (see Supplementary Table 7 for participating centers and inclusion numbers) for all consecutive mCRC patients treated with FTD/TPI was performed between November 2016 and March 2022.<br><br>RECOURSE study<br>Patients were accrued at multiple hospitals globally, as described in the original publication (Mayer et al, NEJM, 2015). All patients were accrued between June 17, 2012, and October 8, 2013.

Outcomes

In the real-world discovery analysis, we searched for genome-wide somatic variants associated with OS and time on FTD/TPI treatment as endpoints. In the real-world validation analysis, the primary and secondary objective was to assess the association of KRAS G12 mutation with OS and PFS, respectively, in the population as a whole and in RAS/RAF mutation-based subpopulations. All endpoints used in real-life analyses were measured from start of FTD/TPI treatment and evaluated at participating institutions over the treatment course according to local practice. In our re-analysis of the RECOURSE trial, we tested OS and PFS benefit of FTD/TPI vs placebo as primary and secondary endpoints, respectively, in subgroups defined by codon-specific KRAS mutation status. This was in accordance with the hierarchy of endpoints prespecified in the RECOURSE trial protocol and these re-analyses were prespecified in a formal data request to the sponsor of the RECOURSE study before access to the data was granted.
