# Supplementary figures and images for: Codon-specific KRAS mutations predict survival benefit of trifluridine/tipiracil in metastatic colorectal cancer
Source: Nat Med. 2023 Mar 2;29(3):605–14. doi: 10.1038/s41591-023-02240-8 (PMC10033412; doi:10.1038/s41591-023-02240-8)

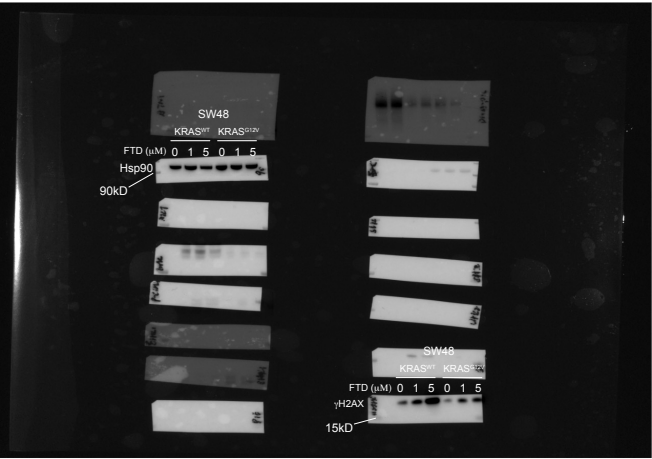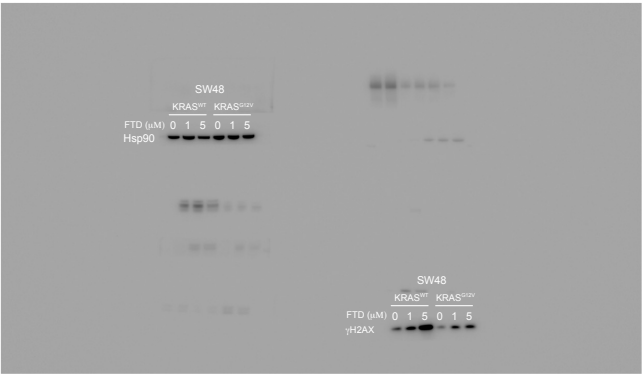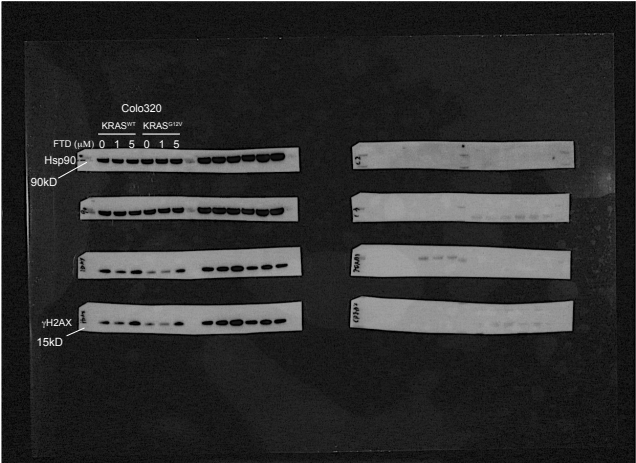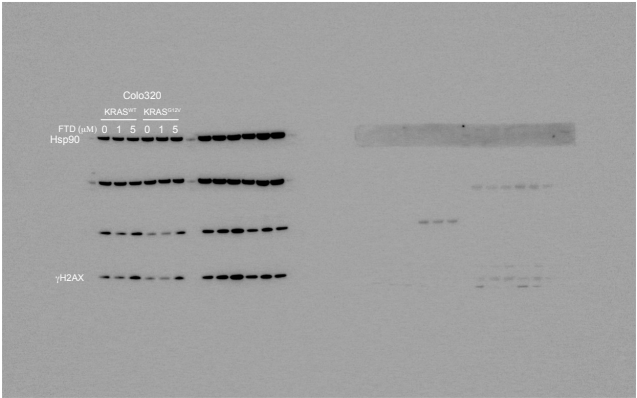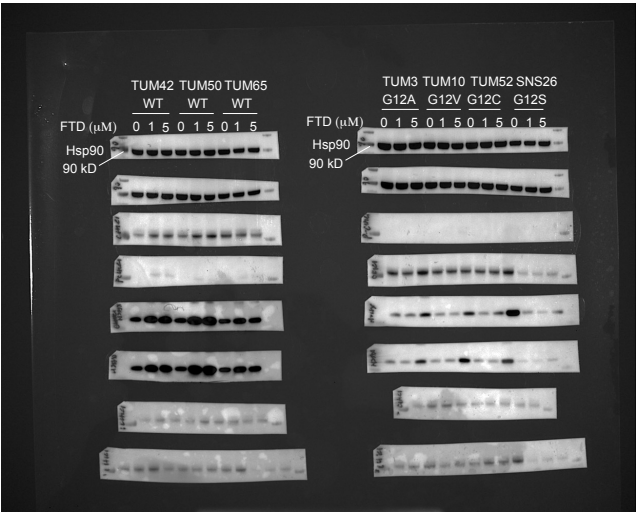

Supplement: Supplementary file 3 — Source data for Fig. 4g,h. [file 41591_2023_2240_MOESM3_ESM.pdf]
